# Enantioselective *de novo* synthesis of 14-hydroxy-6-oxomorphinans†

**DOI:** 10.1039/d4cc01788a

**Published:** 2024-05-10

**Authors:** Jonathan C. Moore, Louis Modell, Jacqueline R. Glenn, Kieran D. Jones, Stephen P. Argent, J. Robert Lane, Meritxell Canals, Hon Wai Lam

**Affiliations:** a The GlaxoSmithKline Carbon Neutral Laboratories for Sustainable Chemistry, University of Nottingham, Jubilee Campus Triumph Road Nottingham NG7 2TU UK hon.lam@nottingham.ac.uk; b School of Chemistry, University of Nottingham, University Park Nottingham NG7 2RD UK; c School of Pharmacy, University of Nottingham Biodiscovery Institute, University of Nottingham Nottingham NG7 2RD UK; d Division of Physiology, Pharmacology and Neuroscience, School of Life Sciences, Queen's Medical Centre, University of Nottingham Nottingham NG7 2UH UK; e Centre of Membrane Protein and Receptors, Universities of Birmingham and Nottingham The Midlands UK

## Abstract

The enantioselective *de novo* synthesis of pharmacologically important 14-hydroxy-6-oxomorphinans is described. 4,5-Desoxynaltrexone and 4,5-desoxynaloxone were prepared using this route and their biological activities against the opioid receptors were measured.

## Graphical abstract

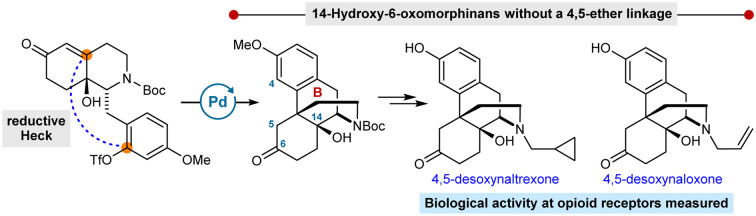

4,5-Epoxymorphinan opioids are important therapeutic compounds.^1^ For example, morphine (1) and codeine (2) are μ-opioid receptor agonists used to treat pain, whereas naloxone (3) and naltrexone (4) are opioid receptor antagonists used to treat opioid overdose and addiction, respectively (Scheme 1A). All four of these compounds are WHO essential medicines.^2^

**Scheme 1:**
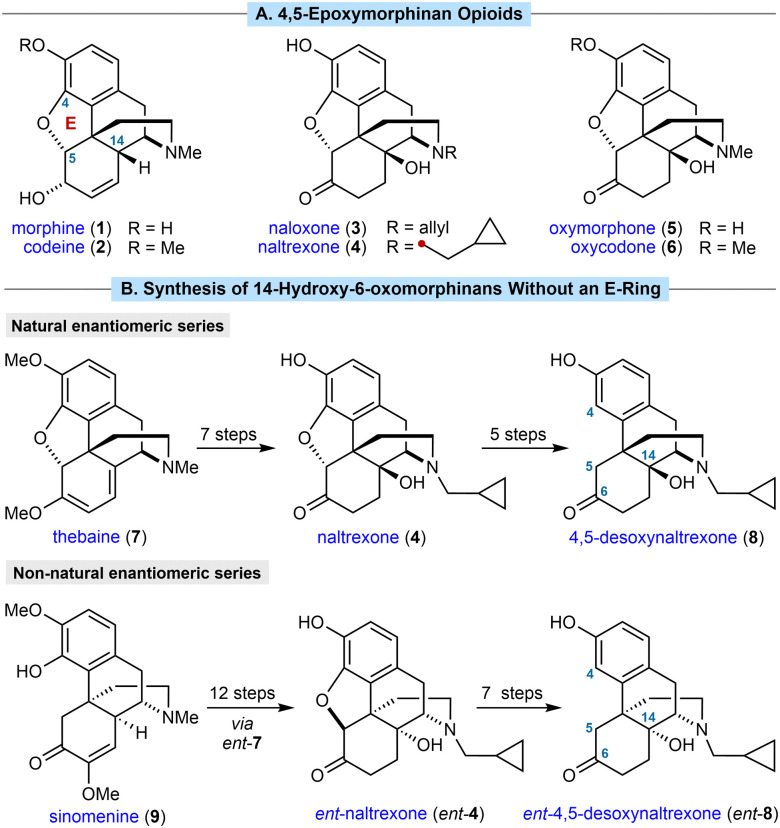
(A) Representative morphinan opioids. (B) Previous methods for the semisynthesis of 14-hydroxy-6-oxomorphinans without an E-ring.

Extensive efforts to discover new morphinans with improved properties have led to a range of compounds being approved for clinical use and a strong understanding of structure–activity relationships (SAR).^1*b*,3^ Introducing a hydroxyl group at C14 has significant effects on both potency and selectivity, and this moiety is present in many prescribed 4,5-epoxymorphinans such as naloxone (3), naltrexone (4), and the analgesics oxymorphone (5) and oxycodone (6). 14-Hydroxy-6-oxomorphinans without an E-ring, so lacking a 4,5-ether linkage (for example, 8 in Scheme 1B), are also of pharmacological interest. They have been shown to exhibit useful bioactivities^4,5^ such as increased κ-opioid receptor (KOR) selectivity relative to their 4,5-epoxy counterparts,^5^ which has been linked with greater efficacy in the treatment of substance abuse.^6^

In common with most other commercially produced morphinan opioids, 14-hydroxy-6-oxomorphinans without a 4,5-ether linkage are prepared from poppy-derived materials, such as thebaine (7). For example, 4,5-desoxynaltrexone (8) can be prepared in five steps by removal of the 4,5-ether linkage of naltrexone (4),^7,8^ which in turn is prepared from thebaine (7) in seven steps (Scheme 1B, top).^9^ Although successful, there are several drawbacks with this approach. First, the sequence is rather lengthy because of the need for several protecting group manipulations to achieve both *N*-demethylation and removal of the 4,5-ether linkage.^7,8^ Second, poppy cultivation is highly space- and resource-intensive, and could be affected by floods, droughts, climate change, disease, and geopolitical events. Illegal poppy diversion also fuels the illicit production and trade of narcotics such as heroin, resulting in huge societal problems. Third, poppy-derived materials exist as a single enantiomeric series, which means they are unsuitable for preparing the non-natural enantiomers of morphinans. For example, *ent*-naltrexone (*ent*-4) and its derivatives, such as *ent*-4,5-desoxynaltrexone (*ent*-8), are antagonists of the Toll-like receptor 4 (TLR4), and have potentially beneficial therapeutic effects such as reversal of neuropathic pain in rat models, and potentiation of morphine analgesia.^9^ However, the preparation of *ent*-4 required 12 steps from the non-poppy-derived natural product sinomenine (9) (Scheme 1B, bottom),^9^ which contrasts with the established seven-step synthesis of naltrexone (4) from thebaine (7).^9^ Seven additional steps were used to convert *ent*-4 into *ent*-4,5-desoxynaltrexone (*ent*-8).^9^ A more direct synthesis of these important compounds would therefore be of high value.

In principle, total synthesis can reduce human reliance on poppy cultivation and enable access to morphinans as their non-natural enantiomers. However, despite extensive research in the total synthesis of morphine and related compounds,^1*b*,10,11^ the *de novo* synthesis of 14-hydroxy-6-oxomorphinans without an E-ring has not, to our knowledge, been described previously. Herein, we describe a concise route for the enantioselective total synthesis of these compounds through the preparation of 4,5-desoxynaltrexone (8) and 4,5-desoxynaloxone (10). The route can also be used to prepare compounds of the non-natural enantiomeric series.

In the semisynthesis of morphinans, installation of the C14 hydroxyl group is achieved *via* oxidation of the 1,3-diene in thebaine (7).^12,13^ Our approach centers around epoxidation of the less-hindered face of the tetrasubstituted alkene 11, followed by a base-promoted β-elimination/epoxide ring-opening to give 12 (Scheme 2A), which has not been described previously in morphinan synthesis.^14^ We anticipated that 12 could then undergo B-ring closure by a reductive Heck cyclization of the aryl triflate onto the enone. However, the use of aryl triflates in Heck reactions for morphinan synthesis has not been reported previously.^15^

**Scheme 2:**
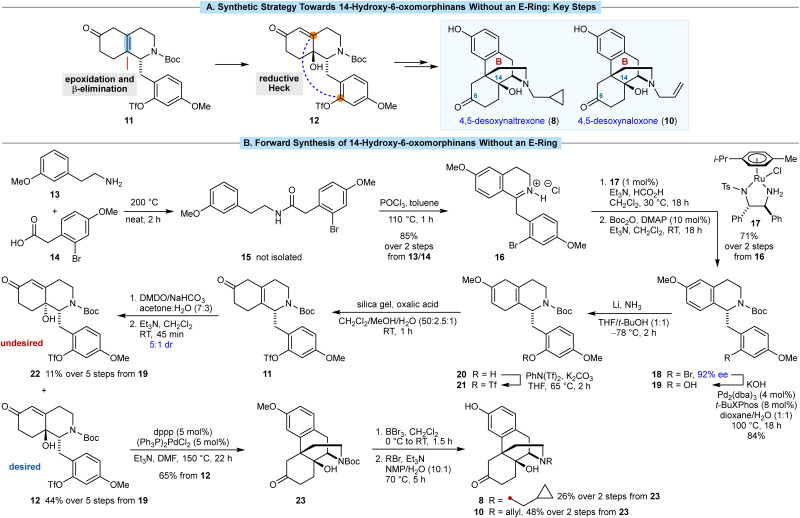
(A) Key synthetic steps. (B) Forward synthesis of 14-hydroxy-6-oxymorphinans without an E-ring.

Our synthesis began with the amide coupling of amine 13 with carboxylic acid 14, both of which are commercially available (Scheme 2B). Heating a mixture 13 and 14 to 200 °C for 2 h in the absence of solvent gave amide 15, which was not isolated. After cooling the mixture to 110 °C, toluene and POCl_3_ were added, and heating was continued for 1 h to promote a Bischler–Napieralski reaction. Evaporation of the solvent and recrystallization of the mixture from MeOH/Et_2_O gave the isoquinolinium hydrochloride salt 16 in 85% yield over the two steps. The corresponding free base of 16 was found to be unstable, and the methylene group adjacent to the 2-bromo-4-methoxyphenyl ring underwent gradual autoxidation to give a ketone. A Noyori reduction of 16 using 1 mol% of RuCl(*p*-cymene)[(*S*,*S*)-Tsdpen] (17) as the catalyst,^16,17^ followed by *N*-Boc protection of the resulting product, gave tetrahydroisoquinoline 18 in 71% yield over two steps and in 92% ee. The aryl bromide in 18 was then converted into the corresponding phenol 19 in 84% yield *via* a palladium-catalyzed reaction with KOH.^18^ This step is necessary to prevent reduction of the arene of the phenol in the Birch reduction through formation of the corresponding lithium phenoxide *in situ*.

The subsequent five steps were then carried out without purification of any intermediates. A chemoselective Birch reduction of 19 gave methyl enol ether 20,^19^ which was converted into aryl triflate 21 with PhN(Tf)_2_ and K_2_CO_3_. Hydrolysis of the methyl enol ether of 21 was successful using aqueous HCl or AcOH in THF or DME, but the desired skipped enone 11 was accompanied by significant isomerization of the alkene to give the corresponding conjugated enone. However, oxalic acid and silica gel^20^ smoothly converted 21 into 11 without competing alkene isomerization.

To install the C14 hydroxyl group, we expected that epoxidation of the tetrasubstituted alkene of 11 would occur on the face opposite to the substituted benzyl group, which upon base-mediated ring-opening of the epoxide by β-elimination, would give 12 with the correct stereochemistry. In the event, it was challenging to find epoxidation conditions that gave both good conversion and high diastereoselectivity (see ESI† for further details). After significant experimentation, we found that addition of a preformed solution of oxone-free DMDO^21^ in acetone/H_2_O (2 : 1) to a solution of 11 in acetone/H_2_O (3 : 1), which was saturated with NaHCO_3_, led to epoxidation with full conversion. Treatment of the crude mixture with Et_3_N to promote β-elimination/epoxide ring-opening then gave a 5 : 1 mixture of alcohols 12 (desired) and 22 (undesired). These diastereomers were readily separated by column chromatography and their relative and absolute configurations were determined by X-ray crystallography.† Following column chromatography, the desired diastereomer 12 was isolated in 44% yield over five steps from 11, which equates to an average yield of 85% per step. The minor diastereomer 22 was also obtained in 11% yield over the five steps.

We then investigated the key reductive Heck cyclization to form the B-ring. The only prior report of a reductive Heck cyclization for construction of the morphinan scaffold was described by the Overman group in 1994.^15*b*^ In that case, the product obtained was not useful for their desired goal of a formal total synthesis of morphine. When the conditions described by Overman^15*b*^ [(Ph_3_P)_2_Pd(TFA)_2_, Et_3_N, toluene, 110 °C] were applied to our substrate 12, no reaction was observed. However, heating 12 in the presence of (Ph_3_P)_2_PdCl_2_ (5 mol%), dppp (5 mol%), and Et_3_N in DMF at 150 °C^22^ for 22 h successfully gave morphinan 23 in 65% yield.

Compound 23 is a versatile precursor to a variety of 14-hydroxy-6-oxomorphinans. Treatment of 23 with BBr_3_ cleaved the aryl methyl ether and Boc group to give 4,5-desoxynoroxymorphone, which was not purified but reacted directly with cyclopropylmethyl bromide to give 4,5-desoxynaltrexone (8) in an unoptimized (attempted once only) 26% yield over two steps. Repeating this sequence but with allyl bromide as the alkylating agent gave 4,5-desoxynaloxone (10) in 48% yield, the structure of which was confirmed by X-ray crystallography. To our knowledge, desoxynaloxone (10) has only been described briefly in the patent literature.^23^

While 4,5-desoxynaltrexone (8) has been shown to bind to the μ-, δ-, and κ-opioid receptors (MOR, DOR and KOR, respectively),^5*a*,8*b*^ its activity (agonist or antagonist) has not been described. The biological activity of 4,5-desoxynaloxone (10) towards the opioid receptors has also not been reported. We therefore assessed the biological activity of 8 and 10 at the four opioid receptor subtypes: MOR, DOR, KOR, and NOP (nociceptin opioid peptide receptor) in HEK293 cells using a bioluminescence resonance energy transfer (BRET) assay, which monitors heterotrimeric G-protein dissociation upon opioid receptor activation. 4,5-Desoxynaltrexone (8) and 4,5-desoxynaloxone (10) show agonist activity at the MOR, DOR, and KOR, with potencies in the low nM range (Table 1). This agonist activity is more pronounced than for naloxone (3) and naltrexone (4), which are used clinically as opioid receptor antagonists (Table 1).^24^ While 4,5-desoxynaltrexone (8) and 4,5-desoxynaloxone (10) displayed robust agonism at the KOR, their agonist activity at the MOR and DOR exhibit lower potencies and maximal effects. As observed with naloxone (3) and naltrexone (4), neither of our test compounds showed agonist or antagonist activity against the NOP.

**Table 1:** Biological activity of 4,5-desoxynaltrexone (8) and 4,5-desoxynaloxone (10) compared with naloxone (3) and naltrexone (4)^a^

	hMOR		hDOR		hKOR		hNOP	
	pEC_50_ (EC_50_, nM)	*E*_max_ (% DAMGO)	pEC_50_ (EC_50_, nM)	*E*_max_ (% SNC-80)	pEC_50_ (EC_50_, nM)	*E*_max_ (% U-50488)	pEC_50_/pIC_50_ (EC_50_/IC_50_, nM)	*E*_max_ (% nociceptin)
8	8.62 ± 0.04 (2.4)	49 ± 5	8.50 ± 0.06 (3.2)	84 ± 4	9.24 ± 0.08 (0.6)	94 ± 4	n/a	n/a
10	8.43 ± 0.05 (3.7)	25 ± 4	7.70 ± 0.07 (20)	68 ± 4	8.59 ± 0.06 (2.6)	83 ± 3	n/a	n/a
3	8.69 ± 0.33 (2.0)	13 ± 2	7.25 ± 0.40 (56)	13 ± 2	7.94 ± 0.14 (12)	64 ± 4	n/a	n/a
4	8.86 ± 0.35 (1.4)	24 ± 4	7.66 ± 0.18 (22)	36 ± 3	8.58 ± 0.13 (2.7)	82 ± 4	n/a	n/a

a Potency (pEC_50_) and maximal effect (*E*_max_) were measured using a G-protein dissociation assay in HEK293 cells expressing human MOR, DOR, KOR, or NOP. EC_50_ is expressed in nM and *E*_max_ as the % of the response elicited by a maximal concentration of reference compounds (DAMGO for MOR, SNC-80 for DOR, U-50488 for KOR, and nociceptin for NOP). The data show mean ± SEM of at least 3 independent experiments performed in duplicate.

In summary, we have described a concise, enantioselective *de novo* synthesis of pharmacologically important 14-hydroxy-6-oxomorphinans without an E-ring, which were previously only prepared through semisynthetic methods using poppy-derived starting materials. 4,5-Desoxynaltrexone (8) and 4,5-desoxynaloxone (10) were prepared in 13 steps (defined as distinct chemical operations) conducted in 11 reaction vessels, starting from inexpensive, commercial compounds 13 and 14. By telescoping several steps in the route, the synthesis of 8 and 10 requires only six chromatographic purifications. The synthesis can be used to prepare the opposite enantiomeric series of 14-hydroxy-6-oxomorphinans by simply using the other enantiomer of chiral catalyst in the Noyori reduction. Finally, the biological activities of 4,5-desoxynaltrexone (8) and 4,5-desoxynaloxone (10) against the opioid receptors were measured, which showed these compounds display partial agonism against the MOR, DOR, and KOR.^25^

This work was supported by the Leverhulme Trust (grant number RPG-2020-150), the Biotechnology and Biological Sciences Research Council (grant number BB/T013966/1), the Engineering and Physical Sciences Research Council (grant number EP/V047124/1), and an Academy of Medical Sciences Professorship Award to M. C. (grant number AMSPR/1013). We thank Alistair Groves (University of Nottingham) for preliminary studies on this project.

## Conflicts of interest

There are no conflicts to declare.

## Supplementary Material

CC-060-D4CC01788A-s001

CC-060-D4CC01788A-s002
