# Supplementary material for: Enantioselective de novo synthesis of 14-hydroxy-6-oxomorphinans
Source: Chem Commun (Camb). 2024 May 10;60(47):6007–10. doi: 10.1039/d4cc01788a (PMC11155717; doi:10.1039/d4cc01788a)
Supplement: CC-060-D4CC01788A-s001 [file CC-060-D4CC01788A-s001.pdf]

Electronic Supplementary Material (ESI) for Chemical Communications.
This journal is © The Royal Society of Chemistry 2024

Enantioselective de novo synthesis of 14-hydroxy-6-oxomorphinans

Jonathan C. Moore,[1,2] Louis Modell,[3] Jacqueline R. Glenn,[4,5] Kieran D. Jones,[1,2] Stephen P. Argent,[2] J. Robert Lane,[4,5] Meritxell Canals,[4,5] and Hon Wai Lam[1,2,*]

[1] The GlaxoSmithKline Carbon Neutral Laboratories for Sustainable Chemistry, University of Nottingham, Jubilee Campus, Triumph Road, Nottingham, NG7 2TU, United Kingdom

[2] School of Chemistry, University of Nottingham, University Park, Nottingham, NG7 2RD, United Kingdom

[3] School of Pharmacy, University of Nottingham Biodiscovery Institute, University of Nottingham, Nottingham, NG7 2RD, United Kingdom

[4] Division of Physiology, Pharmacology and Neuroscience, School of Life Sciences, Queen's Medical Centre, Universityof Nottingham, Nottingham, NG7 2UH, United Kingdom

[5] Centre of Membrane Protein and Receptors, Universities of Birmingham and Nottingham, The Midlands, United Kingdom

Contents

1. General Information 2
2. Final Synthetic Procedures 3
3. Optimization of the Epoxidation of Alkene **11** 15
4. Assessment of the Biological Activity of 4,5-Desoxynaltrexone (**8**) and 4,5-Desoxynaloxone (**10**) Towards the Opioid Receptors and Comparison with Known Opioids 16
5. NMR Spectra 18
6. HPLC Traces 29
7. References 30

1. General Information

Reactions. All air-sensitive reactions were carried out under an inert atmosphere using oven-dried apparatus.

Reagents and Solvents. All commercially available reagents were used as received unless otherwise stated. Petroleum ether refers to Sigma-Aldrich product 24587 (petroleum ether boiling point 40-60 °C).

Chromatography. Thin layer chromatography (TLC) was performed on Merck DF-Alufoilien 60F254 0.2 mm precoated plates. Compounds were visualized by exposure to UV light or by dipping the plates into solutions of potassium permanganate followed by gentle heating. Column chromatography was carried out using a Biotage Isolera 4 fitted with Agela Claricep silica gel disposable flash columns.

Melting Points. Melting points were recorded on a Gallenkamp melting point apparatus and are uncorrected. The solvent of recrystallization is reported in parentheses.

IR Spectra. Infrared (IR) spectra were recorded on Bruker platinum alpha FTIR spectrometer on the neat compound using the attenuated total refraction technique.

NMR Spectra. ^{1}H and ^{13}C NMR spectra were referenced to external tetramethylsilane via the residual protonated solvent (^{1}H) or the solvent itself (^{13}C). All chemical shifts are reported in parts per million (ppm). For $CDCl_3$, the shifts are referenced to 7.26 ppm for ^{1}H NMR spectroscopy and 77.16 ppm for ^{13}C NMR spectroscopy. For DMSO-D_6, the shifts are referenced to 2.50 ppm for ^{1}H NMR spectroscopy and 39.52 ppm for ^{13}C NMR spectroscopy. For CD_3OD, the the shifts are referenced to 3.31 ppm for ^{1}H NMR spectroscopy and 49.00 ppm for ^{13}C NMR spectroscopy. ^{13}C NMR Assignments were made using the DEPT sequence with secondary pulses at 90° and 135° or using 2D NMR spectroscopy techniques including HSQC and HMBC. Coupling constants (J) are quoted to the nearest 0.1 Hz.

Mass Spectra. Electrospray ionisation (ESI) high-resolution mass spectrometry (HRMS) analyses were performed on a Bruker micrOTOFII mass spectrometer (Bruker Daltonik, Bremen, Germany), interfaced to an Agilent 1200 HPLC (Agilent Technologies, Santa Clara, USA). Samples were presented in solution for analysis by Flow Injection, 1 μL of solution being injected into the ion source of the instrument along with a flow of 0.2 mL min^{-1} of 70% MeOH/H_2O eluent. The mass spectrometer was operated in electrospray ionisation (ESI) mode at a typical resolving power of 8000. Control of the analysis was performed through Bruker's Compass Open Access QC automated data acquisition and reporting software (v1.3; Bruker Daltonik, Bremen, Germany).

X-ray Crystallography. Single crystal X-ray diffraction data for compounds **10**, **12**, and **22** were collected on an Oxford Diffraction GV1000 (TitanS2 CCD area detector, mirror-monochromated Cu-Kα radiation source; λ = 1.54184 Å, ω scans). Single crystals were selected, mounted using Fomblin® (YR-1800 perfluoropolyether oil) on a polymer-tipped MiTeGen MicroMount™, and cooled rapidly to 120 K in a stream of cold N_2 using an Oxford Cryosystems open flow cryostat.[1] Cell parameters were refined from the observed positions of all strong reflections and absorption corrections were applied using a Gaussian numerical method with beam profile correction (CrysAlisPro).[2] Structures were solved within Olex2[3] by dual space iterative methods (SHELXT)[4] and all non-hydrogen atoms refined by full-matrix least-squares on all unique F2 values with anisotropic displacement parameters (SHELXL).[5] Hydrogen atoms were refined both freely and with constrained riding geometries and thermal parameters linked to Uiso of their parent atoms. Structures were checked with checkCIF (http://checkcif.iucr.org). CCDC 2341582–2341584 contain the supplementary data for these compounds. These data can be obtained free of charge from The Cambridge Crystallographic Data Centre via www.ccdc.cam.ac.uk/data_request/cif.

2. Final Synthetic Procedures

1-(2-Bromo-4-methoxybenzyl)-6-methoxy-3,4-dihydroisoquinolin-2-ium chloride (16)

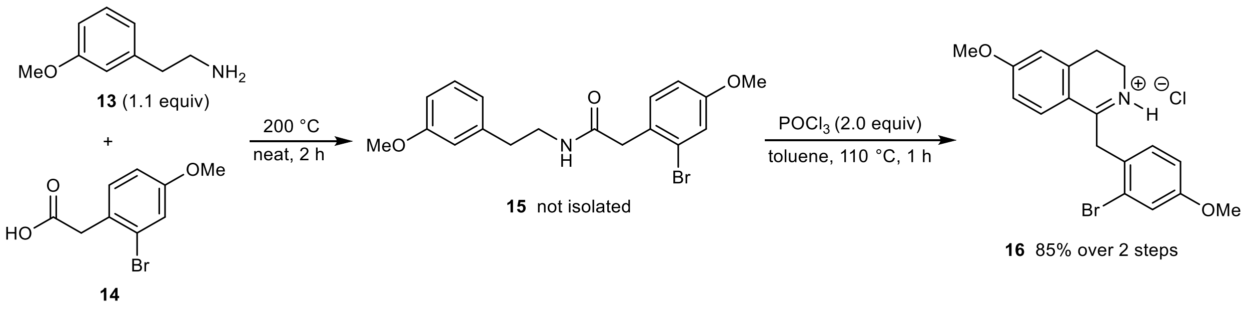

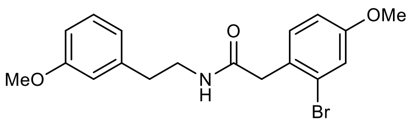

2-(2-Bromo-4-methoxyphenyl)-*N*-(3-methoxyphenethyl)acetamide (15). 2-(2-Bromo-4-methoxyphenyl)acetic acid (**14**) (8.10 g, 33.1 mmol) and 2-(3-methoxyphenyl)ethan-1-amine (**13**) (5.3 mL, 36.5 mmol) were combined in a 500 mL round-bottomed flask fitted with a reflux condenser and the mixture was heated at 200 °C under argon, with stirring. Once the reagents had fully melted, heating was continued for 2 h. **Note:** For the telescoped sequence this mixture was used directly for the next step (vide infra). From a previous run, a purified sample of **15** was obtained by column chromatography (0–40% EtOAc in pentane) for analytical purposes. R_f = 0.21 (30% EtOAc/petroleum ether); m.p. 73–78 °C (Et_2O); IR (ATR) 3318 (N-H), 3069, 3007, 2936, 2835, 1644 (C=O), 1602, 1542, 1490, 1250 (C-O), 1182, 1038 cm^{-1}; ^1H NMR (500 MHz, $CDCl_3$) δ 7.15–7.10 (2H, m, 2 × Ar**H**), 7.07 (1H, d, *J* = 2.6 Hz, Ar**H**), 6.78 (1H, dd, *J* = 8.5,

2.7 Hz, Ar**H**), 6.71 (1H, br ddd, *J* = 8.2, 2.6, 1.0 Hz, Ar**H**), 6.66–6.61 (2H, m, Ar**H**), 5.57 (1H, br t, *J* = 5.8 Hz, N**H**), 3.76 (3H, s, C**H**$_3$), 3.75 (3H, s, C**H**$_3$), 3.57 (2H, s, O=CC**H**$_2$), 3.45 (2H, td, *J* = 6.9, 5.7 Hz, C**H**$_2$N), 2.71 (2H, t, *J* = 6.9 Hz, C**H**$_2$CH$_2$N); ^{13}C NMR (126 MHz, CDCl$_3$) 169.9 (C), 159.8 (C), 159.4 (C), 140.3 (C), 132.0 (CH), 129.6 (CH), 126.7 (C), 125.2 (C), 121.0 (CH), 118.3 (CH), 114.4 (CH), 114.0 (CH), 111.8 (CH), 55.5 (CH$_3$), 55.1 (CH$_3$), 43.1 (CH$_2$), 40.6 (CH$_2$), 35.5 (CH$_2$); HRMS (ESI) Exact mass calculated for [C$_{18}$H$_{21}$^{79}BrNO$_3$]$^+$ [M+H]$^+$: 378.0699, found 378.0700; Exact mass calculated for [C$_{18}$H$_{21}$^{81}BrNO$_3$]$^+$ [M+H]$^+$: 380.0679, found 380.0681.

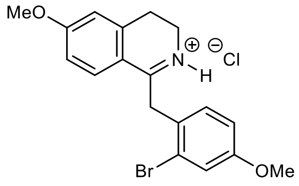

1-(2-Bromo-4-methoxybenzyl)-6-methoxy-3,4-dihydroisoquinolin-2-ium chloride (16). The above reaction mixture was cooled to 110 °C and toluene (100 mL) was added followed by phosphoryl trichloride (6.2 mL, 66.3 mmol). The mixture was heated at 110 °C for 1 h and then concentrated *in vacuo*. The residue was dissolved in MeOH (200 mL) at reflux and then allowed to cool to room temperature. Et$_2$O (80 mL) was added, and the mixture was placed in a –20 °C freezer for 2 days. The resulting crystals were filtered, washed with Et$_2$O, and dried under reduced pressure to give the title compound **16** as a yellow crystalline solid (11.20 g, 85% over two steps). m.p. 219–227 °C (MeOH/Et$_2$O); IR (ATR) 2525, 1907, 1660, 1600, 1566, 1491, 1329, 1262, 1238, 1020 cm^{-1}; ^{1}H NMR (500 MHz, DMSO-D$_6$) δ 12.98 (1H, br s, N**H**), 7.86 (1H, d, *J* = 8.9 Hz, Ar**H**), 7.32 (1H, d, *J* = 8.6 Hz, Ar**H**), 7.27 (1H, d, *J* = 2.6 Hz, Ar**H**), 7.11 (1H, d, *J* = 2.6 Hz, Ar**H**), 7.03 (1H, dd, *J* = 8.9, 2.6 Hz, Ar**H**), 6.97 (1H, dd, *J* = 8.6, 2.7 Hz, Ar**H**), 4.59 (2H, s, N=CC**H**$_2$), 3.89 (3H, s, C**H**$_3$), 3.82 (2H, t, *J* = 7.8 Hz, C**H**$_2$N), 3.77 (3H, s, C**H**$_3$), 3.11 (2H, t, *J* = 7.8 Hz, C**H**$_2$CH$_2$N); ^{13}C NMR (126 MHz, DMSO-D$_6$) δ 174.4 (C), 165.8 (C), 159.5 (C), 141.6 (C), 132.8 (CH), 132.1 (CH), 125.0 (C), 124.5 (C), 118.2 (CH), 117.6 (C), 114.5 (CH), 114.0 (CH), 113.8 (CH), 56.2 (CH$_3$), 55.7 (CH$_3$), 40.9 (CH$_2$), 37.8 (CH$_2$), 24.9 (CH$_2$); HRMS (ESI) Exact mass calculated for [C$_{18}$H$_{19}$^{79}BrNO$_2$]$^+$ [M–Cl]$^+$: 360.0594, found 360.0579; Exact mass calculated for [C$_{18}$H$_{19}$^{81}BrNO$_2$]$^+$ [M–Cl]$^+$: 362.0573, found 362.0578.

tert-Butyl (R)-1-(2-bromo-4-methoxybenzyl)-6-methoxy-3,4-dihydroisoquinoline-2(1H)-carboxylate (18)

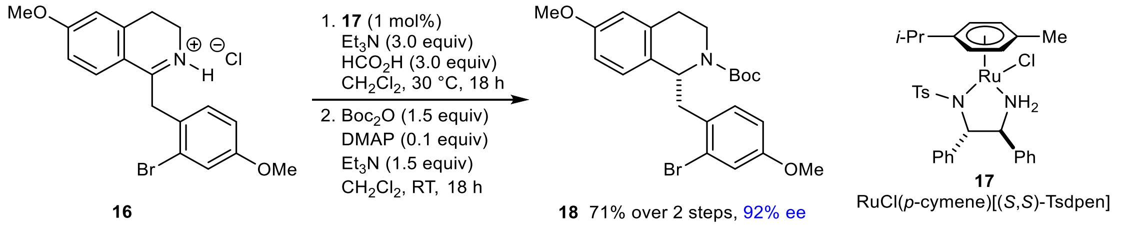

16 **18** 71% over 2 steps, 92% ee **17** RuCl(*p*-cymene)[(*S*,*S*)-Tsdpen]

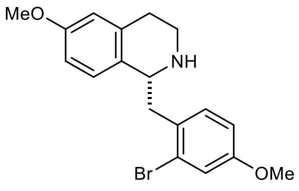

(R)-1-(2-Bromo-4-methoxybenzyl)-6-methoxy-1,2,3,4-tetrahydroisoquinoline (S1). To a suspension of the dihydroisoquinolinium chloride **16** (5.00 g, 12.60 mmol) in CH_2Cl_2 (19 mL) at 0 °C was added HCO_2H (1.4 mL, 37.1 mmol) and then slowly, Et_3N (5.1 mL, 38.1 mmol). The mixture was warmed to warm to room temperature and stirred for 1 h before being warmed to 30 °C. RuCl(*p*-cymene)[(*S,S*)-Ts-DPEN] (**17**, 80 mg, 0.13 mmol) was added and the mixture was evacuated and back-filled with argon three times. After 18 h, the mixture was basified by the addition of saturated aqueous Na_2CO_3 solution and then extracted three times with CH_2Cl_2. The combined organic extracts were dried (Na_2SO_4) and concentrated *in vacuo* to yield the crude title compound **S1**, which was used directly in the next step without further purification.

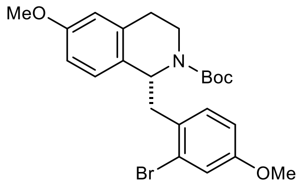

***tert*-Butyl (*R*)-1-(2-bromo-4-methoxybenzyl)-6-methoxy-3,4-dihydroisoquinoline-2(1*H*)-carboxylate (18).** To the above crude mixture was added CH_2Cl_2 (100 mL), Et_3N (5.1 mL, 38.1 mmol), di-*tert*-butyl dicarbonate (4.13 g, 18.9 mmol), and DMAP (154 mg, 1.26 mmol). The reaction was stirred for 18 h and then separated between saturated aqueous NH_4Cl solution and CH_2Cl_2. The aqueous phase was extracted three times with CH_2Cl_2 and the combined organic extracts were dried (Na_2SO_4) and concentrated *in vacuo*. The crude residue was purified by column chromatography (0–20% EtOAc in pentane) to give the title compound **18** as an amorphous white solid (4.11 g, 71% over two steps, 92% ee). **Note**: Peaks in the ^1H and ^{13}C NMR spectra are split as a 4:1 mixture due to the presence of rotamers. Data for only the major rotamer are reported. R_f = 0.46 (30% EtOAc/petroleum ether); $[\alpha]_D^{25}$ −40 (c 1.00, $CHCl_3$); IR (ATR) 2973, 2832, 1674 (C=O), 1602, 1491, 1422, 1235 (C-O), 1164, 1028 (C-N), 854 cm^{-1}; ^1H NMR (500 MHz, $CDCl_3$) δ 7.26 (1H, d, *J* = 8.5 Hz, Ar**H**), 7.14 (1H, d, *J* = 2.6 Hz, Ar**H**), 6.97 (1H, d, *J* = 8.4 Hz, Ar**H**), 6.80 (1H, dd, *J* = 8.7, 3.0 Hz, Ar**H**), 6.78 (1H, dd, *J* = 8.5, 2.7 Hz, Ar**H**), 6.67 (1H, d, *J* = 2.7 Hz, Ar**H**), 5.35 (1H, dd, *J* = 10.9, 3.4 Hz, NC**H**), 4.36 (1H, ddd, *J* = 13.4, 6.1, 2.2 Hz, C**H**$_a$H$_b$N), 3.80 (3H, s, C**H**$_3$), 3.78 (3H, s, C**H**$_3$), 3.27 (1H, ddd, *J* = 13.3, 11.8, 4.0 Hz, CH$_a$**H**$_b$N), 3.19 (1H, dd, *J* = 14.0, 3.4 Hz, CHC**H**$_a$H$_b$),

3.06–2.88 (2H, m, $CHCH_a\mathbf{H}_b$ and $C\mathbf{H}_aH_bCH_2N$), 2.71 (1H, ddd, *J* = 16.3, 4.0, 2.2 Hz, $CH_a\mathbf{H}_bCH_2N$), 1.13 (9H, s, $C(C\mathbf{H}_3)_3$); ^{13}C NMR (126 MHz, $CDCl_3$) 159.1 (C), 158.3 (C), 154.5 (C), 135.9 (C), 132.1 (CH), 130.4 (C), 129.7 (C), 128.2 (CH), 125.5 (C), 118.3 (CH), 113.5 (CH), 113.3 (CH), 112.9 (CH), 79.5 (C), 55.8 (CH_3), 55.5 (CH_3), 54.0 (CH), 42.1 (CH_2), 36.2 (CH_2), 29.1 (CH_2), 28.1 (3 × CH_3); HRMS (ESI) Exact mass calculated for $[C_{23}H_{29}{}^{79}BrNO_4]^+$ $[M+H]^+$: 462.1275, found 462.1273; Exact mass calculated for $[C_{23}H_{29}{}^{81}BrNO_4]^+$ $[M+H]^+$: 464.1254, found 462.1258 Enantiomeric excess was determined by HPLC using a Chiralcel OD-H column (90:10 isohexane: *i*-PrOH, 1.0 mL/min, 230 nm, 25 °C); t_r (minor) = 5.6 min, t_r (major) = 6.6 min, 92% ee.

An authentic racemic sample of **18** for the HPLC assay was obtained by Boc-protection of racemic **S1**, which was itself obtained by reduction of the corresponding free base of dihydroisoquinolinium hydrochloride **16**.

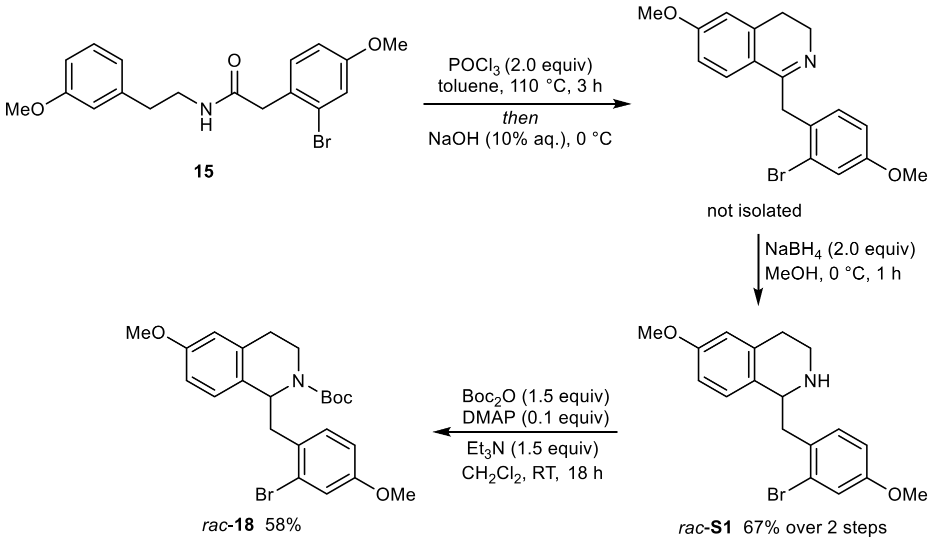

tert-Butyl (*R*)-1-(2-hydroxy-4-methoxybenzyl)-6-methoxy-3,4-dihydroisoquinoline-2(1*H*)-carboxylate (19)

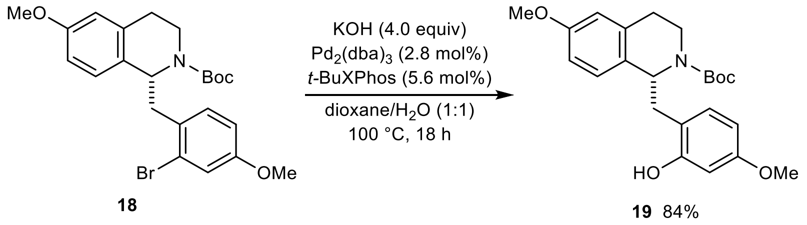

Tetrahydroquinoline **18** (4.11 g, 8.89 mmol), $Pd_2(dba)_3$ (228 mg, 0.249 mmol), *t*-BuXPhos (211 mg, 0.498 mmol), and KOH (1.40 g, 24.9 mmol) were combined in a 250 mL round-bottomed flask fitted with a reflux condenser and evacuated and backfilled with argon three times. 1,4-Dioxane (43 mL) and then H_2O (43 mL, previously been degassed by sonication under high vacuum) were added. The mixture was heated to 100 °C and stirred for 18 h, and then separated between H_2O and CH_2Cl_2. The aqueous phase was extracted three times with CH_2Cl_2 and the combined organic extracts were dried

(Na_2SO_4), and concentrated *in vacuo*. The crude residue was purified by column chromatography (0–20% EtOAc in pentane) to yield the title compound **19** as an amorphous white solid (4.11 g, 71%). **Note**: Peaks in the ^{1}H and ^{13}C NMR spectra are split as a 4:1 mixture due to the presence of rotamers. Data for only the major rotamer are reported. R_f = 0.43 (30% EtOAc/petroleum ether); $[\alpha]_D^{25}$ –28 (c 1.00, $CHCl_3$); IR (ATR) 3234 (O-H), 2931, 1646 (C=O), 1598, 1429, 1367, 1289, 1239 (C-O), 1163, 1038 cm^{-1}; ^{1}H NMR (400 MHz, CD_3OD) δ 7.13 (1H, d, *J* = 8.5 Hz, Ar**H**), 6.82 (1H, d, *J* = 8.2 Hz, Ar**H**), 6.76 (1H, dd, *J* = 8.6, 2.7 Hz, Ar**H**), 6.68 (1H, d, *J* = 2.4 Hz, Ar**H**), 6.39 (1H, d, *J* = 2.4 Hz, Ar**H**), 6.30 (1H, dd, *J* = 8.2, 2.5 Hz, Ar**H**), 5.30 (1H, dd, *J* = 10.5, 3.7 Hz, NC**H**), 4.12 (1H, ddd, *J* = 13.5, 6.1, 2.6 Hz, C$\mathbf{H}_a$$H_b$N), 3.76 (3H, s, OC$\mathbf{H}_3$), 3.72 (3H, s, OC$\mathbf{H}_3$), 3.36 (1H, ddd, *J* = 13.5, 11.2, 4.6 Hz, NC$H_a$$\mathbf{H}_b$), 3.03 (1H, dd, *J* = 13.7, 3.7 Hz, CHC$\mathbf{H}_a$$H_b$), 2.89–2.66 (3H, m, CHC$H_a$$\mathbf{H}_b$ and C$\mathbf{H}_2$$CH_2$N), 1.16 (9H, s, C(C$\mathbf{H}_3$)$_3$); ^{13}C NMR (101 MHz, CD_3OD) δ 161.1 (C), 159.6 (C), 158.0 (C), 156.6 (C), 136.5 (C), 132.6 (CH), 131.3 (C), 129.2 (CH), 119.3 (C), 114.3 (CH), 113.5 (CH), 105.1 (CH), 102.3 (CH), 80.7 (C), 55.8 (CH), 55.6 (2 × CH_3), 37.9 (CH_2), 37.4 (CH_2), 29.8 (CH_2), 28.3 (3 × CH_3); HRMS (ESI) Exact mass calculated for $[C_{23}H_{30}NO_5]^+$ $[M+H]^+$: 400.2119, found 400.2115.

***tert*-Butyl (1*R*,8a*R*)-8a-hydroxy-1-(4-methoxy-2-{[(trifluoromethyl)sulfonyl]oxy}benzyl)-6-oxo-3,4,6,7,8,8a-hexahydroisoquinoline-2(1*H*)-carboxylate (22) and *tert*-butyl (1*R*,8a*S*)-8a-hydroxy-1-(4-methoxy-2-{[(trifluoromethyl)sulfonyl]oxy}benzyl)-6-oxo-3,4,6,7,8,8a-hexahydroisoquinoline-2(1*H*)-carboxylate (12)**

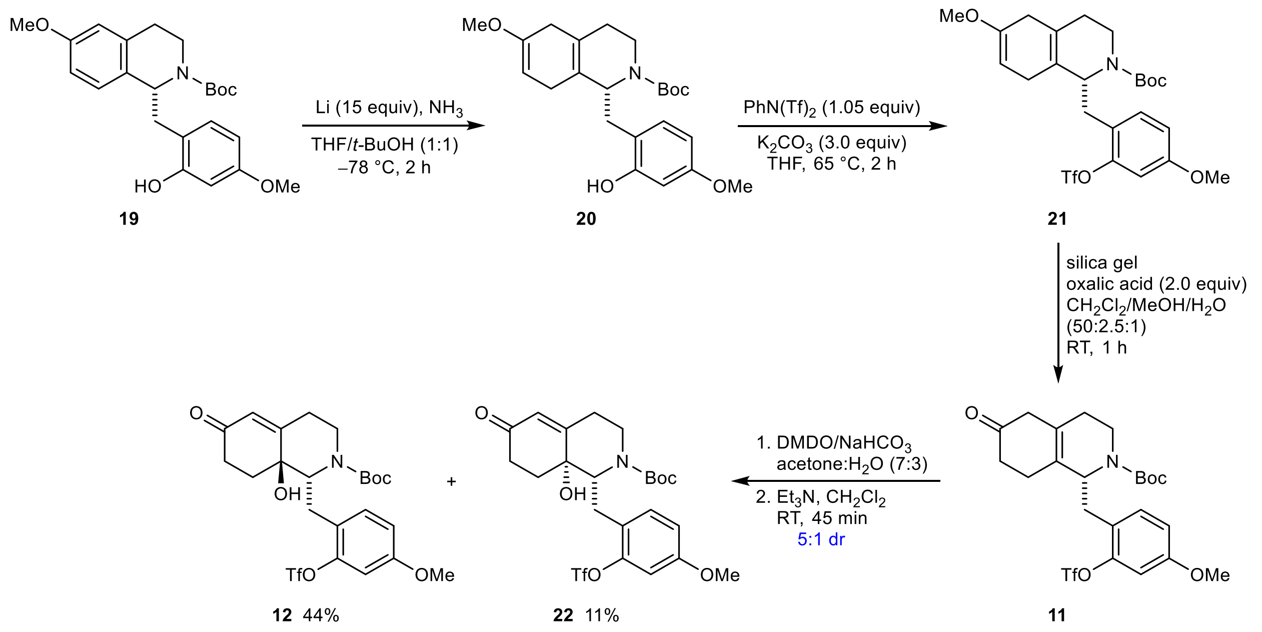

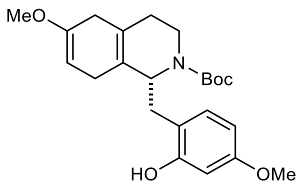

***tert*-Butyl (*R*)-1-(2-hydroxy-4-methoxybenzyl)-6-methoxy-3,4,5,8-tetrahydroisoquinoline-2(1*H*)-carboxylate (20).** In a 100 mL, two-necked round bottomed flask fitted with a dry ice condenser was added the phenol **19** (1.20 g, 3.00 mmol) followed by THF (15 mL) and *t*-BuOH (15 mL). Dry ice and acetone were placed in the condenser and ammonia (*ca.* 30 mL) was condensed into the solution. The mixture was placed in a –78 °C cooling bath and lithium metal (312 mg, 45.0 mmol) was added portionwise over 30 min. The reaction was stirred at –78 °C for 2.5 h and then concentrated by warming to room temperature and sparging with a stream of nitrogen. The residue was separated between CH_2Cl_2 and H_2O and the aqueous phase was extracted three times with CH_2Cl_2. The combined organic phases were dried (Na_2SO_4) and concentrated *in vacuo* to give the crude 1,4-diene **20**, which was used directly in the next step without further purification. Characteristic spectroscopic data for **20**: ^{1}H NMR (500 MHz, $CDCl_3$) δ 6.88 (1H, d, *J* = 8.2 Hz), 6.42 (1H, br s), 6.34 (1H, d, *J* = 8.4 Hz); HRMS (ESI) Exact mass calculated for $[C_{23}H_{31}NNaO_5]^+$ $[M+Na]^+$: 424.2094, found 424.2106.

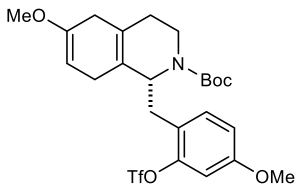

***tert*-Butyl (*R*)-6-methoxy-1-(4-methoxy-2-{[(trifluoromethyl)sulfonyl]oxy}benzyl)-3,4,5,8-tetrahydroisoquinoline-2(1*H*)-carboxylate (21).** To a solution of the crude diene **20** in THF (25 mL) at room temperature was added $PhN(Tf)_2$ (1.13 g, 3.15 mmol) and then K_2CO_3 (1.24 g, 8.97 mmol). The mixture was heated at 65 °C for 2 h, cooled to room temperature, and filtered through cotton wool. The solids were washed with CH_2Cl_2 and the combined filtrates were concentrated *in vacuo* to give the crude aryl triflate **21** as a 2:1 mixture rotamers, which was used directly in the next step without further purification. Characteristic spectroscopic data for **21**: ^{1}H NMR (500 MHz, $CDCl_3$) δ 4.73–4.69 (1H, m, major rotamer), 4.69–4.65 (0.5H, m, minor rotamer), 4.55 (0.5H, d, *J* = 10.4 Hz, minor rotamer), 4.40 (1H, d, *J* = 11.0 Hz, major rotamer), 4.23 (1H, dd, *J* = 13.4, 6.4 Hz, major rotamer), 3.97 (0.5H, dd, *J* = 13.6, 6.2 Hz, minor rotamer); HRMS (ESI) Exact mass calculated for $[C_{24}H_{30}F_3NNaO_7S]^+$ $[M+Na]^+$: 556.1587, found 556.1589.

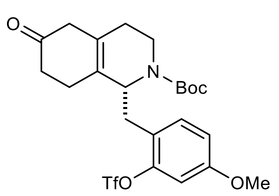

***tert*-Butyl (*R*)-1-(4-methoxy-2-{[(trifluoromethyl)sulfonyl]oxy}benzyl)-6-oxo-3,4,5,6,7,8-hexahydroisoquinoline-2(1*H*)-carboxylate (11).** To a solution of the crude aryl triflate **11** in CH_2Cl_2 (6.0 mL) was added a solution of oxalic acid (540 mg, 6.00 mmol) in MeOH (2.4 mL), silica gel (166 mg), and H_2O (118 µL, 3.00 mmol). The reaction was stirred for 1 h and then filtered through cotton wool. The solids were washed with CH_2Cl_2 and the combined filtrates were washed with 5% NaOH

solution. The aqueous layer was extracted three times with CH_2Cl_2 and the combined organic phases were dried (Na_2SO_4) and concentrated *in vacuo* to give the crude ketone **11** as a *ca.* 1.7:1 mixture of rotamers, which was used directly in the next step without further purification. Characteristic spectroscopic data for **11**: ^{1}H NMR (500 MHz, $CDCl_3$) δ 4.65 (0.6H, d, *J* = 10.1 Hz, minor rotamer), 4.47 (1H, d, *J* = 11.0 Hz, major rotamer), 4.26 (1H, dd, *J* = 13.5, 6.4 Hz, major rotamer), 4.01 (0.6H, dd, *J* = 13.7, 6.3 Hz, minor rotamer); HRMS (ESI) Exact mass calculated for $[C_{23}H_{28}F_3NNaO_7S]^+$ $[M+Na]^+$: 542.1431, found 542.1429.

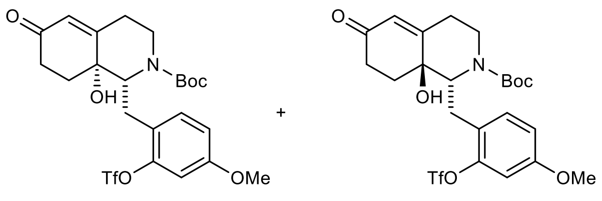

22 **12**

***tert*-Butyl (1*R*,8a*R*)-8a-hydroxy-1-(4-methoxy-2-{[(trifluoromethyl)sulfonyl]oxy}benzyl)-6-oxo-3,4,6,7,8,8a-hexahydroisoquinoline-2(1*H*)-carboxylate (22) and *tert*-butyl (1*R*,8a*S*)-8a-hydroxy-1-(4-methoxy-2-{[(trifluoromethyl)sulfonyl]oxy}benzyl)-6-oxo-3,4,6,7,8,8a-hexahydroisoquinoline-2(1*H*)-carboxylate (12).** To a solution of the crude ketone **11** in acetone (45 mL) and H_2O (15 mL) at room temperature was added $NaHCO_3$ (12.0 g, 143 mmol). The mixture was cooled to 0 °C and then a solution of Oxone-free DMDO (4.5 mmol) in acetone:H_2O (2:1, 90 mL) was added over 20 min. (**Note**: The solution of DMDO used was prepared by adding H_2O to an Oxone-free solution of DMDO in acetone, which was itself prepared according to an existing literature procedure.[6]) The mixture was stirred for 18 h and then partially concentrated to remove most of the acetone. The mixture was extracted seven times with CH_2Cl_2 and the combined organic phases were dried (Na_2SO_4) and concentrated *in vacuo* to yield 1.34 g of crude material. The remaining aqueous phase was an emulsion, which was left to stand for 2 days before brine was added. This aqueous mixture was extracted three times with EtOAc, dried (Na_2SO_4), and concentrated *in vacuo* to give a further 293 mg of crude material. Each of the above crude mixtures were separately dissolved in CH_2Cl_2 (10 mL and 5 mL, respectively) and Et_3N (0.5 mL and 0.25 mL, respectively) was added at room temperature. The reaction mixtures were stirred for 45 min and then concentrated *in vacuo* to yield the crude allyl alcohols **22** and **12** (**22**:**12** = 1:5). The crude mixtures were separately purified by column chromatography (20–80% EtOAc in pentane) to give **22** (172 mg, 11%) followed by **12** (706 mg, 44%) as the combined yields from the two columns, each as off-white amorphous solids.

Data for **22**: **Note**: Peaks in the ^{1}H and ^{13}C NMR spectra are broad due to the presence of rotamers. R_f = 0.32 (50% EtOAc/petroleum ether); m.p. 117–119 °C (EtOAc/pentane); $[\alpha]_D^{25}$ –27 (c 1.00, $CHCl_3$); IR (ATR) 3393 (O-H), 2925, 1661 (C=O), 1624, 1508, 1413, 1366, 1243 (C-O), 1208 (C-O), 1161, 1138 (C-O) cm^{-1}; ^{1}H NMR (500 MHz, CD_3OD) δ 7.28 (1H, d, *J* = 8.6 Hz, Ar**H**), 7.02–6.88 (1H, m, Ar**H**), 6.88–6.73 (1H, m, Ar**H**), 5.85 (1H, d, *J* = 1.9 Hz, O=CC**H**=), 4.51–4.35 (1H, br m,

NC**H**), 3.99–3.73 (1H, br m, C**H**$_a$H$_b$N), 3.81 (3H, s, OC**H**$_3$), 3.71–3.57 (1H, br m, CH$_a$**H**$_b$N), 3.12−3.00 (2H, m, CHC**H**$_2$), 2.88–2.79 (1H, m, C**H**$_a$H$_b$CH$_2$N), 2.65–2.51 (2H, m, CH$_a$**H**$_b$CH$_2$N and O=CC**H**$_a$H$_b$), 2.50–2.26 (2H, m, O=CCH$_a$**H**$_b$ and C**H**$_a$H$_b$COH), 2.15 (1H, ddd, *J* = 13.7, 10.4, 4.9 Hz, CH$_a$**H**$_b$COH), 1.29–0.99 (9H, m, C(C**H**$_3$)$_3$); ^{13}C NMR (126 MHz, CD$_3$OD) δ 200.3 (C), 165.9 (C), 161.1 (C), 156.6 (C), 150.4 (C), 134.8 (CH), 128.1 (CH), 125.1 (C), 120.0 (q, *J* = 319.1 Hz, C), 115.0 (CH), 108.4 (CH), 81.2 (C), 71.3 (C), 61.6 (CH), 56.4 (CH$_3$), 38.5 (CH$_2$), 35.3 (2 × CH$_2$), 31.0 (CH$_2$), 29.0 (CH$_2$), 28.2 (3 × CH$_3$); HRMS (ESI) Exact mass calculated for [$C_{23}H_{28}F_3NNaO_8S$]$^+$ [M+Na]$^+$: 558.1380, found 558.1372.

Recrystallization of **22** from EtOAc/pentane using the vapour diffusion method gave crystals that were suitable for X-ray crystallography:

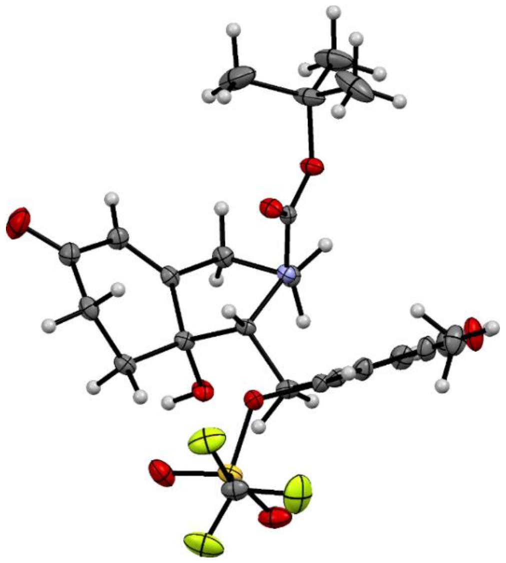

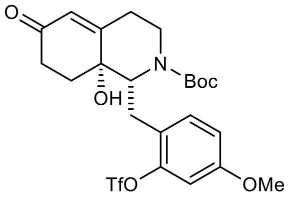

22
CCDC 2341583
Flack parameter –0.006(4)

Data for **12**: **Note**: Peaks in the ^{1}H and ^{13}C NMR spectra are split as a 2.3:1 mixture due to the presence of rotamers. Both rotamers are reported for the ^{1}H NMR data and only the major rotamer is reported for the ^{13}C NMR data.

R$_f$ = 0.21 (50% EtOAc/petroleum ether); m.p. 180 °C (decomposed, EtOAc/pentane); $[\alpha]_D^{25}$ –19 (c 1.00, CHCl$_3$); IR (ATR) 3413 (O-H), 2925, 1667 (C=O), 1624 (C=O), 1509, 1416, 1366, 1317, 1212 (C-O), 1162, 1139, 1068 cm^{-1}; ^{1}H NMR (500 MHz, CD$_3$OD) δ 7.34 (0.3H, d, *J* = 8.6 Hz, Ar**H**, minor rotamer), 7.30 (0.7H, d, *J* = 8.6 Hz, Ar**H**, major rotamer), 6.95 (0.7H, dd, *J* = 8.6, 2.5 Hz, Ar**H**, major rotamer), 6.92 (0.3H, dd, *J* = 8.6, 2.6 Hz, Ar**H**, minor rotamer), 6.84 (0.7H, d, *J* = 2.5 Hz, Ar**H**, major rotamer), 6.80 (0.3H, d, *J* = 2.5 Hz, Ar**H**, minor rotamer), 5.95–5.99 (1H, m, O=CC**H**=, both rotamers), 4.71 (0.3H, dd, *J* = 12.3, 4.3 Hz, NC**H**, minor rotamer), 4.56 (0.7H, dd, *J* = 12.7, 3.7 Hz, NC**H**, major rotamer), 4.11 (0.7H, dddd, *J* = 13.5, 6.6, 2.3, 1.1 Hz, C**H**$_a$H$_b$N, major rotamer), 3.98 (0.3H, dddd, *J* = 13.6, 6.6, 2.7, 1.1 Hz, C**H**$_a$H$_b$N, minor rotamer), 3.81 (2.1H, s, OC**H**$_3$, major rotamer), 3.79 (0.9H, s, OC**H**$_3$, minor rotamer), 3.22–3.04 (2H, m, CH$_a$**H**$_b$N and CHC**H**$_a$CH$_b$, both

rotamers), 2.92–2.79 (1H, m, $C\mathbf{H}_aH_bCH_2N$, both rotamers), 2.78–2.66 (2H, m, $CHCH_aC\mathbf{H}_b$ and $O{=}CC\mathbf{H}_aCH_b$, both rotamers), 2.49–2.29 (3H, m, $CH_a\mathbf{H}_bCH_2N$, $O{=}CCH_aC\mathbf{H}_b$, and $C\mathbf{H}_aH_bCOH$, both rotamers), 2.14–2.04 (1H, m, $CH_a\mathbf{H}_bCOH$, both rotamers), 1.25 (2.7H, s, $C(C\mathbf{H}_3)_3$, minor rotamer), 1.23 (6.3H, s, $C(C\mathbf{H}_3)_3$, major rotamer); ^{13}C NMR (126 MHz, CD_3OD) 200.9 (C), 161.3 (C), 161.1 (C), 157.1 (C), 150.0 (C), 134.0 (CH), 128.2 (CH), 123.7 (C), 120.0 (q, $J_{C\text{-}F}$ = 319.0 Hz, C), 115.0 (CH), 108.8 (CH), 81.3 (C), 71.3 (C), 63.0 (CH), 56.4 (CH_3), 38.4 (CH_2), 34.9 (CH_2), 33.2 (CH_2), 31.0 (CH_2), 29.1 (CH_2), 28.3 (3 × CH_3); HRMS (ESI) Exact mass calculated for $[C_{23}H_{28}F_3NNaO_8S]^+$ $[M+Na]^+$: 558.1380, found 558.1370.

Recrystallization of **12** from EtOAc/pentane using the vapour diffusion method gave crystals that were suitable for X-ray crystallography:

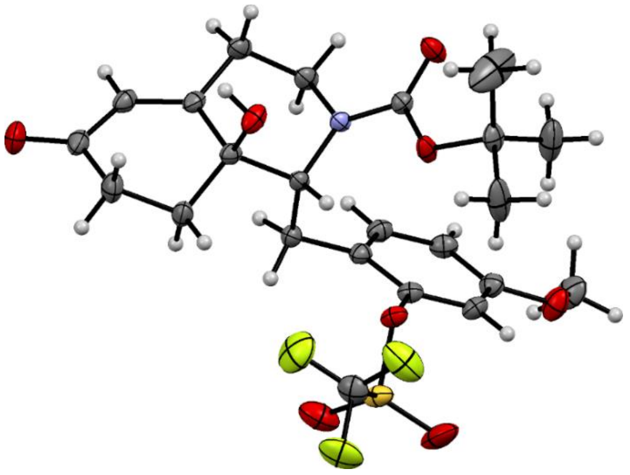

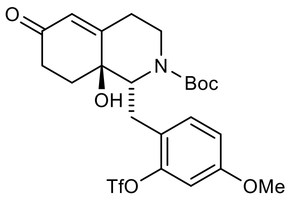

12
CCDC 2341582
Flack parameter 0.004(4)

***tert*-Butyl (4b*R*,8a*S*,9*R*)-8a-hydroxy-3-methoxy-6-oxo-6,7,8,8a,9,10-hexahydro-5*H*-9,4b-(epiminoethano)phenanthrene-11-carboxylate (23)**

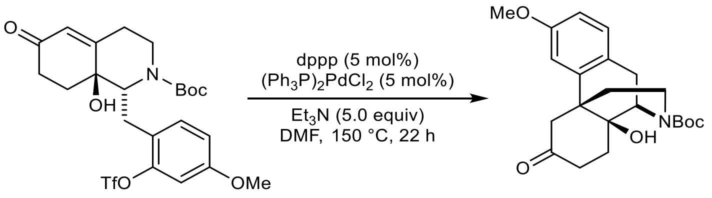

12 **23** 65%

$PdCl_2(PPh_3)_2$ (6.5 mg, 9.3 µmol) and dppp (3.9 mg, 9.5 µmol) were combined in a microwave vial, which was evacuated and backfilled with argon three times. DMF (3.7 mL) was added, and the mixture was stirred for 5 min. The aryl triflate **12** (100 mg, 0.187 mmol) was added followed by Et_3N (125 µL, 0.933 mmol). The vessel was resealed, and the reaction mixture was placed on a 125 °C heating block and stirred for 48 h, cooled to room temperature, and concentrated *in vacuo*. The crude mixture was purified by column chromatography (0–40% EtOAc in cyclohexane) to give the title compound **23** (47 mg, 65%) as an off-white amorphous solid. R_f = 0.28 (50% EtOAc/petroleum ether); $[\alpha]_D^{25}$ −136.0 (c 1.00, $CHCl_3$); IR (ATR) 3426 (O-H), 2971, 2921, 1686 (C=O), 1664 (C=O),

1611, 1417, 1365, 1256 (C-O), 1160 (C-O) cm^{-1}; ^{1}H NMR (500 MHz, $CDCl_3$) δ 6.99 (1H, d, *J* = 8.4 Hz, Ar**H**), 6.81 (1H, d, *J* = 2.6 Hz, Ar**H**), 6.72 (1H, dd, *J* = 8.4, 2.6 Hz, Ar**H**), 4.60–4.19 (1H, br m, C**H**N), 3.90–3.74 (1H, br m, C**H**$_a$H$_b$N), 3.76 (3H, s, OC**H**$_3$), 3.21 (1H, dd, *J* = 18.4, 6.5 Hz, C**H**$_a$H$_b$CHN), 3.00 (1H, d, *J* = 14.3 Hz, O=CC**H**$_a$H$_b$C, 2.89 (1H, d, *J* = 18.3 Hz, CH$_a$**H**$_b$CHN), 2.82 (1H, d, *J* = 14.3 Hz, O=CCH$_a$**H**$_b$C), 2.78 (1H, ddd, *J* = 14.5, 13.0, 7.4 Hz, O=CC**H**$_a$H$_b$CH$_2$), 2.67 (1H, s, O**H**), 2.71-2.53 (1H, br m, CH$_a$**H**$_b$N), 2.22–2.08 (1H, m, C**H**$_a$H$_b$CH$_2$N), 2.13 (1H, ddt, *J* = 14.5, 4.7, 2.1 Hz, O=CCH$_a$**H**$_b$CH$_2$), 1.90 (1H, td, *J* = 12.4, 11.5, 4.2 Hz, O=CCH$_2$C**H**$_a$H$_b$), 1.87–1.82 (1H, m, O=CCH$_2$CH$_a$**H**$_b$), 1.48 (9H, s, C(C**H**$_3$)$_3$), 1.22–1.06 (1H, br m, CH$_a$**H**$_b$CH$_2$N); ^{13}C NMR (126 MHz, $CDCl_3$) δ 209.4 (C), 158.8 (C), 156.7 (C), 139.6 (C), 129.0 (CH), 126.2 (C), 113.1 (CH), 111.5 (CH), 80.7 (C), 69.7 (C), 55.4 (CH_3), 54.0 (CH), 46.1 (CH_2), 45.5 (C), 37.5 (2 x CH_2), 35.5 (CH_2), 32.8 (CH_2), 32.4 (CH_2), 28.5 (3 × CH_3); HRMS (ESI) Exact mass calculated for $[C_{22}H_{29}NNaO_5]^+$ $[M+Na]^+$: 410.1938, found 410.1934.

Desoxynaltrexone

(4b*R*,8a*S*,9*R*)-11-(Cyclopropylmethyl)-3,8a-dihydroxy-8,8a,9,10-tetrahydro-5H-9,4b-(epiminoethano)phenanthren-6(7*H*)-one (8)

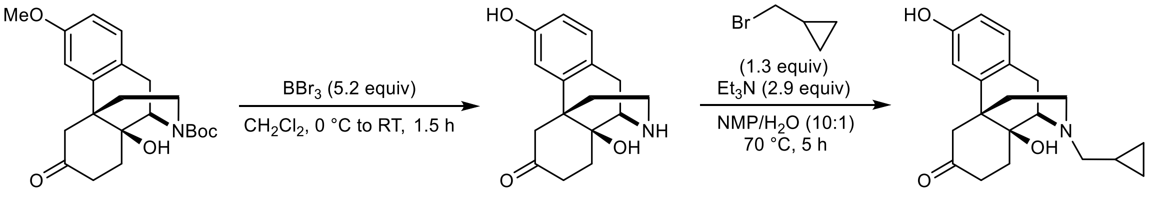

23 4,5-desoxynoroxymorphone 4,5-desoxynaltrexone (**8**)

To a solution of **23** (82 mg, 0.21 mmol) in CH_2Cl_2 (2.1 mL) at 0 °C was added BBr_3 (0.06 mL, 0.63 mmol). The mixture was warmed to room temperature, stirred for 1.5 h, and cooled to 0 °C. The reaction was basified with aqueous NH_4OH solution (30–33% NH_3 in H_2O). The mixture was extracted with three times with $CHCl_3$ and the combined organic phases were dried (Na_2SO_4), filtered, and concentrated *in vacuo* to leave 4,5-desoxynoroxymorphone, which was used directly in the next step without further purification.

To a solution of 4,5-desoxynoroxymorphone in NMP/H_2O (10:1, 0.39 mL) at room temperature was added was (bromomethyl)cyclopropane (27 µL, 0.28 mmol) followed by Et_3N (0.04 mL, 0.30 mmol). The reaction vessel was purged with argon and then placed on a 70 °C heating block for 3 h. Following a further addition of Et_3N (0.04 mL, 0.30 mmol), the mixture was stirred at 70 °C for an additional 17 h. The reaction was cooled to room temperature, diluted with toluene and washed three times with a saturated aqueous $NaHCO_3$ solution. The aqueous phase was extracted with toluene and the combined organic phases were dried (Na_2SO_4), filtered, and concentrated *in vacuo*. The crude residue was purified by column chromatography [0–4% of *solution A* in CH_2Cl_2, where *solution A* was made up by adding MeOH to aqueous NH_3 solution (30%) give a 2 M solution of NH_3] to yield 4,5-

desoxynaltrexone (**8**) as an amorphous white solid (18 mg, 26% over two steps). R_f = 0.16 (5% MeOH/CH_2Cl_2); $[\alpha]_D^{25}$ −140.0 (c 1.00, $CHCl_3$); IR (ATR) 3377 (O-H), 2922, 2832, 1703 (C=O), 1612, 1506, 1447, 1310, 1280, 1232 (C-O) cm^{-1}; ^{1}H NMR (500 MHz, $CDCl_3$) δ 6.94 (1H, d, *J* = 8.2 Hz, Ar**H**), 6.79 (1H, d, *J* = 2.6 Hz, Ar**H**), 6.65 (1H, dd, *J* = 8.3, 2.6 Hz, Ar**H**), 3.14 (1H, d, *J* = 6.4 Hz, C**H**N), 3.07 (1H, d, *J* = 14.1 Hz, O=CC$\mathbf{H}_a$$H_b$C), 3.05 (1H, d, *J* = 18.4 Hz, C$\mathbf{H}_a$$H_b$CHN), 2.88-2.70 (3H, m, C$H_a$$\mathbf{H}_b$CHN, O=CC$H_a$$\mathbf{H}_b$C and O=C$CH_2C\mathbf{H}_a$$H_b$), 2.63-2.56 (1H, m, CH_2C$\mathbf{H}_a$$H_b$N), 2.43-2.38 (2H, m, NC$\mathbf{H}_2$CH), 2.20-2.08 (3H, m, C$\mathbf{H}_a$$H_b$$CH_2N, CH_2CH_a$$\mathbf{H}_b$N and O=C$CH_2CH_a$$\mathbf{H}_b$), 1.91-1.77 (2H, m, O=CC$\mathbf{H}_2$$CH_2$), 1.21-1.16 (1H, m, C$H_a$$\mathbf{H}_b$$CH_2$N), 0.92-0.82 (1H, m, N$CH_2$C**H**), 0.59-0.50 (2H, m, cyclopropyl C$\mathbf{H}_a$$H_bC\mathbf{H}_a$$H_b$), 0.17-0.10 (2H, m, cyclopropyl C$H_a$$\mathbf{H}_bCH_a$$\mathbf{H}_b$); ^{13}C NMR (126 MHz, $CDCl_3$) δ 211.5 (C), 154.8 (C), 140.7 (C), 128.9 (CH), 126.9 (C), 114.3 (CH), 112.7 (CH), 69.1 (C), 59.9 (CH), 59.5 (CH_2), 46.6 (CH_2), 45.8 (C), 43.5 (CH_2), 37.7 (CH_2), 37.0 (CH_2), 32.0 (CH_2), 24.6 (CH_2), 9.6 (CH), 4.1 (CH_2), 4.0 (CH_2); HRMS (ESI) Exact mass calculated for $[C_{20}H_{26}NO_3]^+$ $[M+H]^+$: 328.1907, found 328.1908.

These data are consistent with those reported previously.[7]

4,5-Desoxynaloxone

(4b*R*,8a*S*,9*R*)-11-Allyl-3,8a-dihydroxy-8,8a,9,10-tetrahydro-5*H*-9,4b-(epiminoethano)phenanthren-6(7*H*)-one (10)

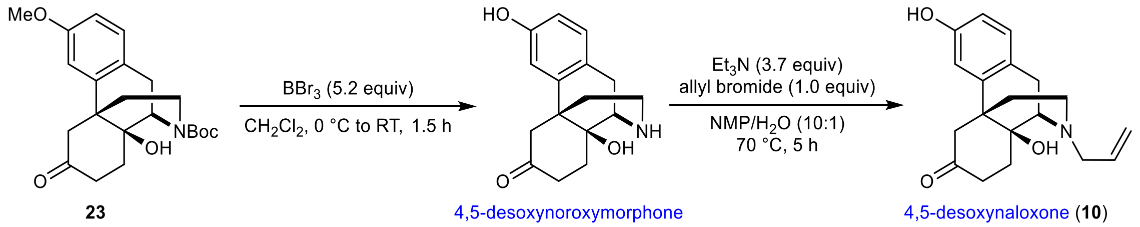

To a solution of **23** (47 mg, 0.12 mmol) in CH_2Cl_2 (1.2 mL) at 0 °C was added BBr_3 (0.06 mL, 0.63 mmol). The mixture was warmed to room temperature, stirred for 1.5 h, and cooled to 0 °C. The reaction was basified with aqueous NH_4OH solution (30–33% NH_3 in H_2O). The mixture was extracted with three times with $CHCl_3$ and the combined organic phases were dried (Na_2SO_4), filtered, and concentrated *in vacuo* to leave 4,5-desoxynoroxymorphone, which was used directly in the next step without further purification.

To a solution of 4,5-desoxynoroxymorphone in NMP/H_2O (10:1, 0.39 mL) at room temperature was added was allyl bromide (10.7 µL, 0.124 mmol) followed by Et_3N (0.03 mL, 0.22 mmol). The reaction vessel was purged with argon and then placed on a 70 °C heating block for 2 h. Following a further addition of Et_3N (0.03 mL, 0.22 mmol), the mixture was stirred at 70 °C for an additional 3 h. The reaction was cooled to room temperature, diluted with CH_2Cl_2 and washed three times with a saturated aqueous $NaHCO_3$ solution. The aqueous phase was extracted with CH_2Cl_2 and the combined

organic phases were dried (Na_2SO_4), filtered, and concentrated *in vacuo*. The crude residue was purified by column chromatography (0–8% of *solution A* in CH_2Cl_2, where *solution A* was made up by adding MeOH to aqueous NH_3 solution (30%) give a 2 M solution of NH_3) to yield 4,5-desoxynaloxone (**10**) as a white solid (14 mg, 48% over two steps). R_f = 0.19 (5% MeOH/CH_2Cl_2); m.p. 222-225 °C (decomposed, EtOAc/pentane); $[\alpha]_D^{25}$ −128.0 (c 1.00, $CHCl_3$); IR (ATR) 3385 (O-H), 2924, 2843, 1701 (C=O), 1612, 1503, 1446, 1309, 1280, 1229 (C-O) cm^{-1}; ^1H NMR (500 MHz, CD_3OD) δ 6.96 (1H, d, J = 8.3 Hz, Ar**H**), 6.69 (1H, d, J = 2.5 Hz, Ar**H**), 6.59 (1H, dd, J = 8.3, 2.5 Hz, Ar**H**), 5.89 (1H, ddt, J = 16.7, 10.2, 6.4 Hz, C**H**=CH_2), 5.24 (1H, app dq, J = 17.2, 1.7 Hz, =C**H**$_a$H$_b$), 5.16 (1H, app dq, J = 10.2, 1.3 Hz, =CH$_a$**H**$_b$), 3.22 (1H, ddt, J = 13.6, 6.5, 1.4 Hz, C**H**$_a$H$_b$CH=CH_2), 3.17 (1H, ddt, J = 13.6, 6.3, 1.4 Hz, CH$_a$**H**$_b$CH=CH_2), 3.13 (1H, d, J = 18.6 Hz, C**H**$_a$H$_b$CHN), 3.02 (1H, d, J = 14.1 Hz, O=CC**H**$_a$H$_b$C), 2.99 (1H, d, J = 6.4 Hz, C**H**N), 2.81-2.70 (3H, m, CH$_a$**H**$_b$CHN, O=CCH$_a$**H**$_b$C, and O=CCH$_2$C**H**$_a$H$_b$), 2.54-2.48 (1H, m, CH$_2$C**H**$_a$H$_b$N), 2.21-2.08 (2H, m, C**H**$_a$H$_b$CH$_2$N and CH$_2$CH$_a$**H**$_b$N), 2.05 (1H, ddt, J = 14.6, 5.3, 2.0 Hz, O=CCH$_2$CH$_a$**H**$_b$), 1.87 (1H, td, J = 13.5, 5.3 Hz, O=CC**H**$_a$H$_b$CH$_2$), 1.77 (1H, ddd, J = 13.5, 7.2, 1.9 Hz, O=CCH$_a$**H**$_b$CH$_2$), 1.14 (1H, dt, J = 11.4, 2.2 Hz, CH$_a$**H**$_b$CH$_2$N); ^{13}C NMR (126 MHz, CD_3OD) δ 213.1 (C), 157.1 (C), 141.6 (C), 137.0 (CH), 129.7 (CH), 127.4 (C), 118.1 (CH_2), 115.1 (CH), 113.2 (CH), 70.4 (C), 61.2 (CH), 58.7 (CH_2), 47.1 (CH_2), 46.6 (C), 44.3 (CH_2), 38.3 (CH_2), 37.9 (CH_2), 33.0 (CH_2), 25.4 (CH_2); HRMS (ESI) Exact mass calculated for $[C_{19}H_{24}NO_3]^+$ $[M+H]^+$: 314.1751, found 314.1752.

Recrystallization of **10** from EtOAc/pentane using the vapour diffusion method gave crystals that were suitable for X-ray crystallography:

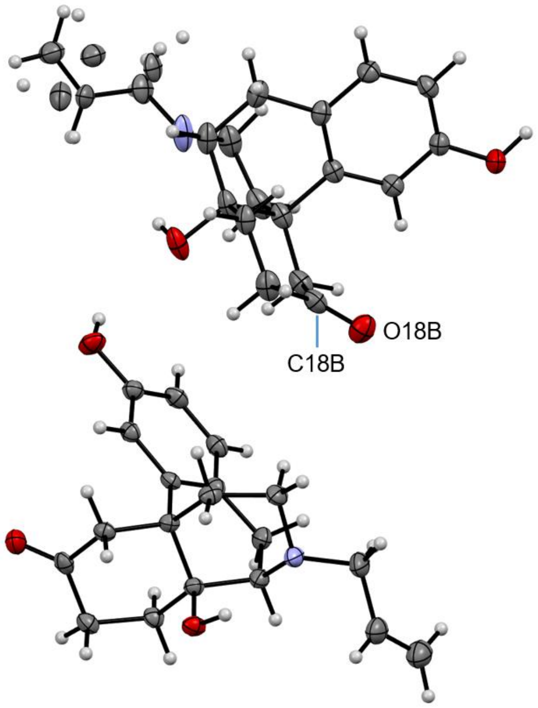

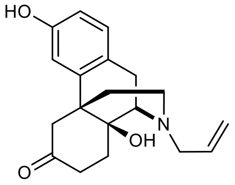

4,5-desoxynaloxone (**10**)
CCDC 2341584
Flack parameter −0.05(3)

Note: There are two crystallography independent molecules in the asymmetric unit. In one of these molecules, there is disorder associated with the allyl group. A residual electron density peak with height 0.67 e $Å^{-3}$ is observed in the electron density map 1.01 and 1.41 Å from oxygen atom O18B and carbon atom C17B respectively. The atom can be modelled as an oxygen atom with a partial occupancy fraction of 0.08; however, no sensible disorder model in agreement with the bulk analysis data for this compound could be developed.

3. Optimization of the Epoxidation of Alkene 11

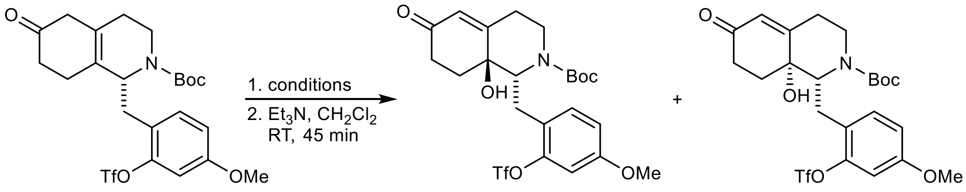

11 **12** **22**

Table S1. Optimization of epoxidation of **11**

Reagent(s)	Solvent	dr (**12**:**22**)	Conv. (%)
m-CPBA	toluene	1:3	>95
m-CPBA	CH_2Cl_2	1:2	>95
m-CPBA	MeCN	1.3:1	>95
Oxone/$NaHCO_3$	acetone/H_2O (2:1)	5:1	~50
DMDO	CH_2Cl_2/acetone (1:4)	2:1	>95
DMDO/$NaHCO_3$	acetone/H_2O (2:1)	5:1	>95

Epoxidation of **11** with *m*-CPBA in toluene and β-elimination/epoxide ring-opening with Et_3N gave the desired 14-hydroxyenone in good yield but as a 1:3 mixture of diastereomers in favor of the undesired isomer **22** (Table S1). Increasing the polarity of the solvent in the epoxidation step by changing from toluene to CH_2Cl_2 and then to MeCN led to more of the desired isomer **12** being formed. In MeCN, the ratio of **12**:**22** was 1.3:1. Changing the oxidant to dimethyldioxirane (DMDO), which was generated *in situ* by the reaction of acetone with Oxone in the presence of $NaHCO_3$, increased the diastereoselectivity to 5:1 in favor of **12**. However, this reaction stalled at around 50% conversion. Despite efforts to push the reaction to completion by increasing the reaction time, increasing the equivalents of oxidant and base, or with slower addition of Oxone, considerable quantities of starting material **11** always remained. It is known that DMDO can be decomposed by its reaction with Oxone[8] and it seemed plausible that this side-reaction was consuming the DMDO before full consumption of the alkene **11** occurred. Accordingly, conducting the epoxidation in CH_2Cl_2/acetone (1:4) with a preformed solution of DMDO in acetone (in the absence of Oxone) led to full consumption of **11**. In this case, however, the diastereoselectivity dropped to 2:1. However, by repeating this reaction but changing the solvent system back to acetone/H_2O (2:1), which was saturated with $NaHCO_3$, full consumption of **11** was achieved, and after β-elimination/epoxide ring-opening with Et_3N, **12** was obtained with 5:1 dr (see page 9 for the detailed experimental procedure).

4. Assessment of the Biological Activity of 4,5-Desoxynaltrexone (8) and 4,5-Desoxynaloxone (10) Towards the Opioid Receptors and Comparison with Known Opioids

The activity of 4,5-desoxynaltrexone (**8**) and 4,5-desoxynaloxone (**10**) at opioid receptors was tested using a Bioluminescence Resonance Energy Transfer (BRET) assay that monitors G protein dissociation upon receptor activation. For comparison, the activity of naloxone (**3**) and naltrexone (**4**) were also measured in parallel using the same assay. Unless otherwise stated, reagents were purchased from Sigma Aldrich-Merck. Experiments were performed in transiently transfected human embryonic kidney 293T (HEK 293T) cells as described previously.[9] Briefly, cells were cultured at 37 °C, 5 % CO_2 in Dulbecco's modified eagle medium (DMEM) supplemented with 10 % (v/v) fetal bovine serum (FBS). Cells were seeded in 10 cm Petri dishes (3 x 10^6 cells per dish) and allowed to grow overnight in full media at 37 °C, 5% CO_2. Cells were transiently transfected the next day, in media supplemented with antibiotics (100 U/mL penicillin and 100 µg/mL streptomycin, Gibco) using a 1:6 total DNA to PEI (PolySciences Inc) ratio. Transfected constructs were as follows: 1 µg of opioid receptor (MOR, DOR, KOR or NOP), 2 µg of WT-Gα_{i2}, 1 µg of Gβ1-Venus(156-239), 1 µg of Gγ2-Venus(1-155), 1 µg of masGRK3ct-Rluc8. The following day, cells were plated in Greiner poly-D-lysine-coated, white bottom 96-well plates (SLS) in full media. On the day of the assay (48h post-transfection), cells were washed once with D-PBS (Lonza, SLS) and incubated in D-PBS for 30 min at 37 °C. The Rluc substrate coelenterazine h (NanoLight) was added to each well (final concentration of 5 µM) and ligands (final concentration from 10 µM to 0.01 nM in D-PBS) were added to the wells before reading the plate at 37 °C in a PHERAstar FSX microplate reader (Venus and Rluc emission signals at 535 and 475 nm, respectively, BMG Labtech) every minute for 10 min. The ratio between Venus fluorescence and Rluc luminescence (BRET ratio) was used to quantify the BRET signal in each well. Data were normalized to maximal and minimal response of the corresponding reference agonist (DAMGO for MOR, SNC-80 for DOR, U-50488 for KOR and nociceptin for NOP). All data points represent the mean of at least three independent experiments performed in duplicate. Data were fitted using the built-in log(agonist) vs. response (three parameters) model in Prism 9.0 (GraphPad software Inc., San Diego, CA) to obtain values of potency (pEC_{50}) and maximal effect (Emax).

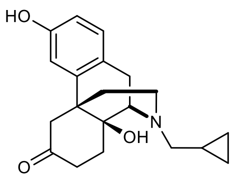

4,5-desoxynaltrexone (**8**)

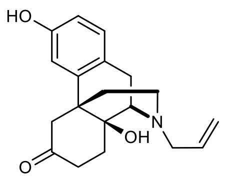

4,5-desoxynaloxone (**10**)

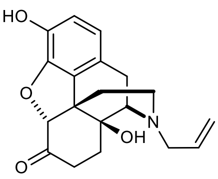

naloxone (**3**)

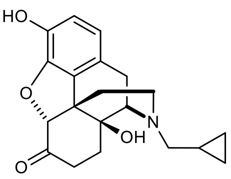

naltrexone (**4**)

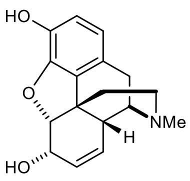

morphine (**1**)

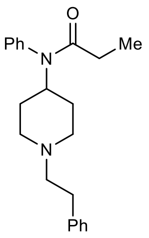

fentanyl

	hMOR		hDOR		hKOR		hNOP	
	pEC_{50} (EC_{50}, nM)	Emax (% DAMGO)	pEC_{50} (EC_{50}, nM)	Emax (% SNC-80)	pEC_{50} (EC_{50}, nM)	Emax (% U-50488)	pEC_{50}/pIC_{50} (EC_{50}/IC_{50}, nM)	Emax (% nociceptin)
**8**	8.62 ± 0.04 (2.4)	49 ± 5	8.50 ± 0.06 (3.2)	84 ± 4	9.24 ± 0.08 (0.6)	94 ± 4	n/a	n/a
**10**	8.43 ± 0.05 (3.7)	25 ± 4	7.70 ± 0.07 (20)	68 ± 4	8.59 ± 0.06 (2.6)	83 ± 3	n/a	n/a
**3**	8.69 ± 0.33 (2.0)	13 ± 2	7.25 ± 0.40 (56)	13 ± 2	7.94 ± 0.14 (12)	64 ± 4	n/a	n/a
**4**	8.86 ± 0.35 (1.4)	24 ± 4	7.66 ± 0.18 (22)	36 ± 3	8.58 ± 0.13 (2.7)	82 ± 4	n/a	n/a
morphine (**1**)	7.89 ± 0.07 (13)	106 ± 4						
fentanyl	8.9 ± 0.11 (1.3)	103 ± 7						

[a] Potency (pEC_{50}) and maximal effect (Emax) were measured using a G-protein dissociation assay in HEK293 cells expressing human MOR, DOR, KOR, or NOP. EC_{50} is expressed in nM and Emax as the % of the response elicited by a maximal concentration of reference compounds (DAMGO for MOR, SNC-80 for DOR, U-50488 for KOR, and nociceptin for NOP). The data show mean ± SEM of at least 3 independent experiments performed in duplicate.

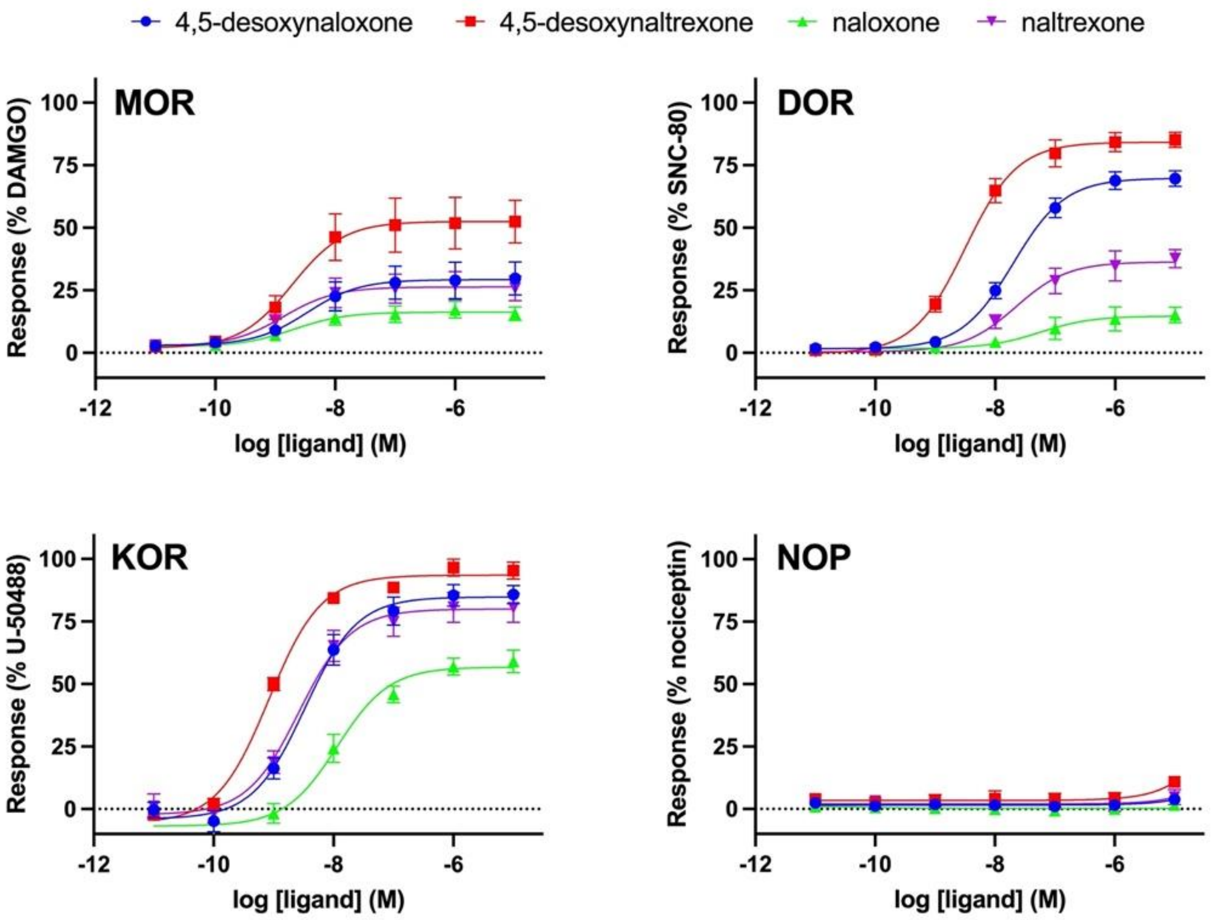

5. NMR Spectra

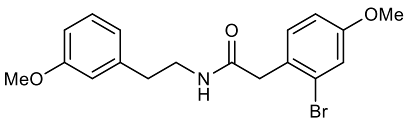

15

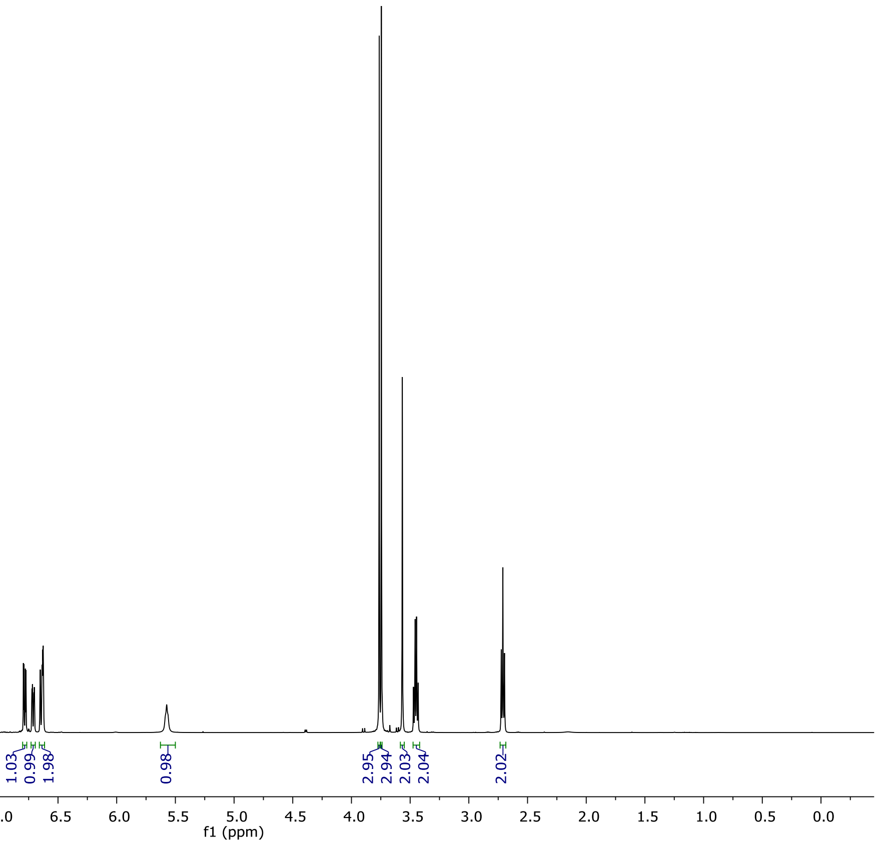

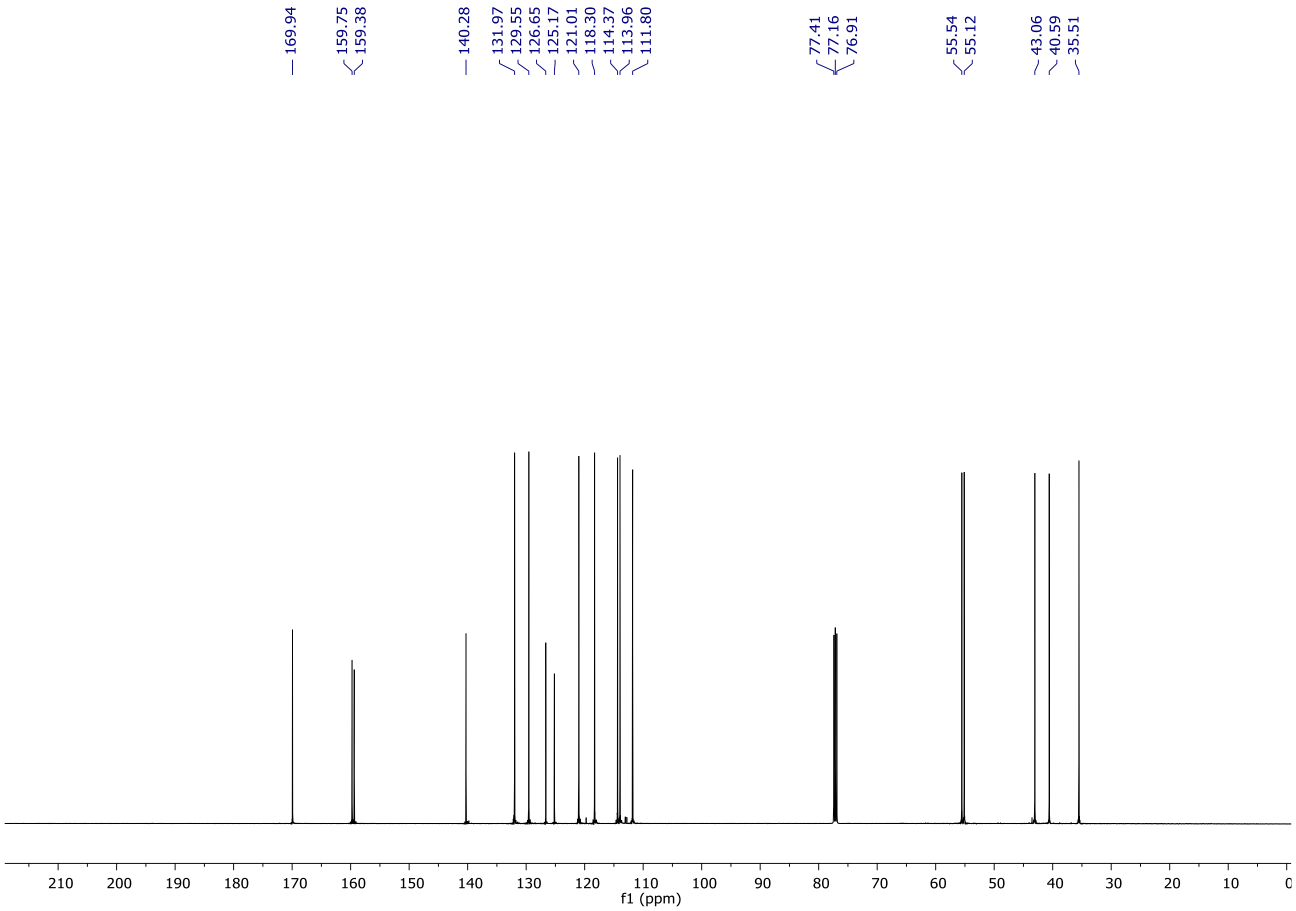

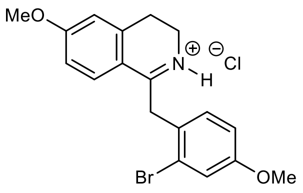
MeO
N
H
Cl
Br
OMe

16

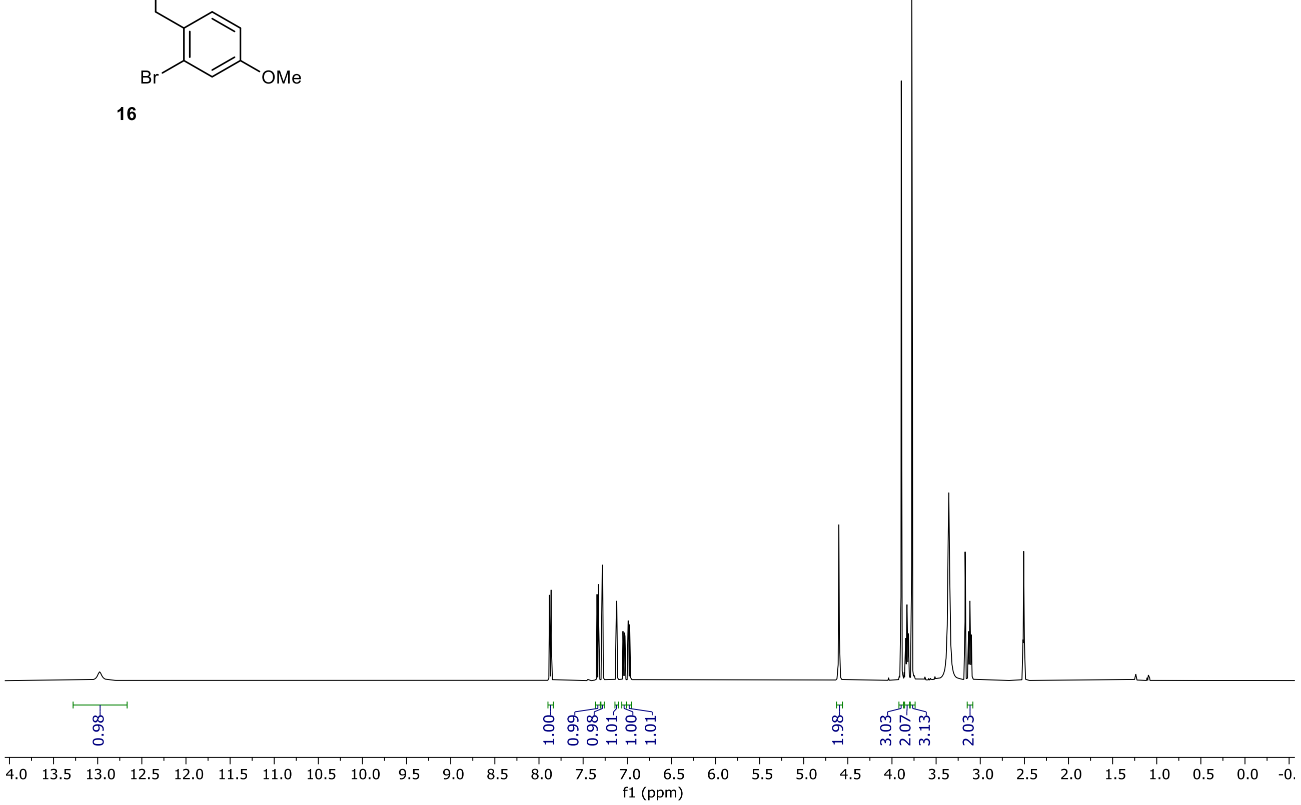
0.98
1.00
0.99
0.98
1.01
1.00
1.01
1.98
3.03
2.07
3.13
2.03
f1 (ppm)

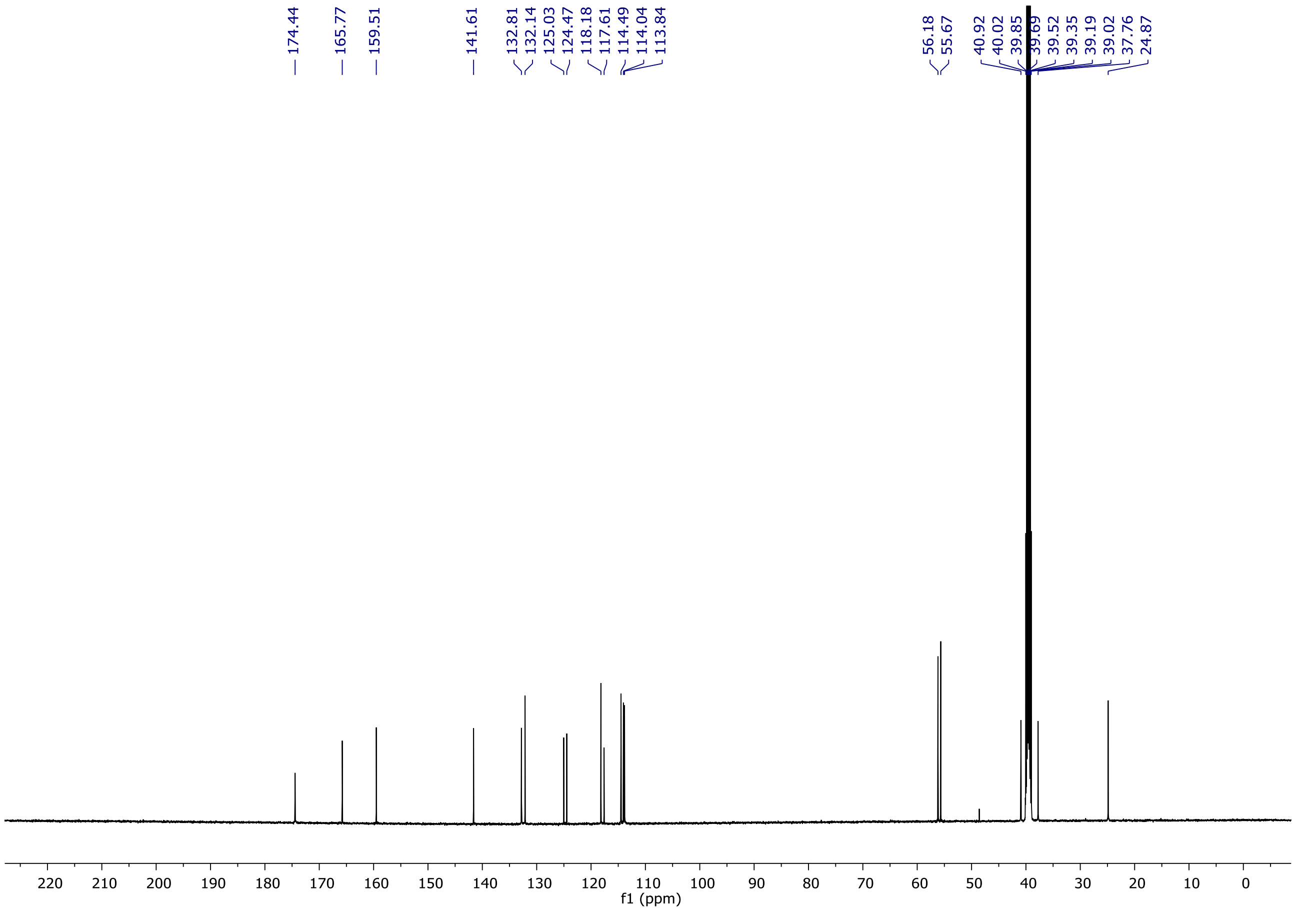
174.44
165.77
159.51
141.61
132.81
132.14
125.03
124.47
118.18
117.61
114.49
114.04
113.84
56.18
55.67
40.92
40.02
39.85
39.69
39.52
39.35
39.19
39.02
37.76
24.87
f1 (ppm)

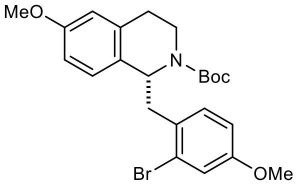
MeO
N
Boc
Br
OMe

18

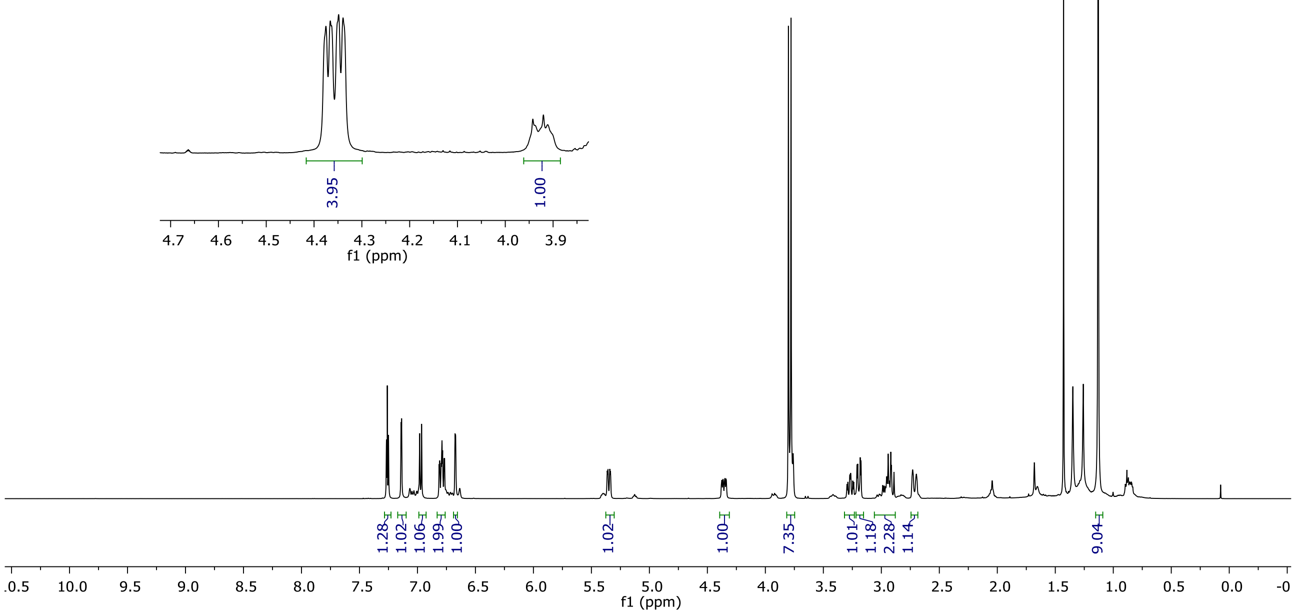
3.95
1.00
4.7 4.6 4.5 4.4 4.3 4.2 4.1 4.0 3.9
f1 (ppm)
1.28
1.02
1.06
1.99
1.00
1.02
1.00
7.35
1.01
1.18
2.28
1.14
9.04
10.5 10.0 9.5 9.0 8.5 8.0 7.5 7.0 6.5 6.0 5.5 5.0 4.5 4.0 3.5 3.0 2.5 2.0 1.5 1.0 0.5 0.0
f1 (ppm)

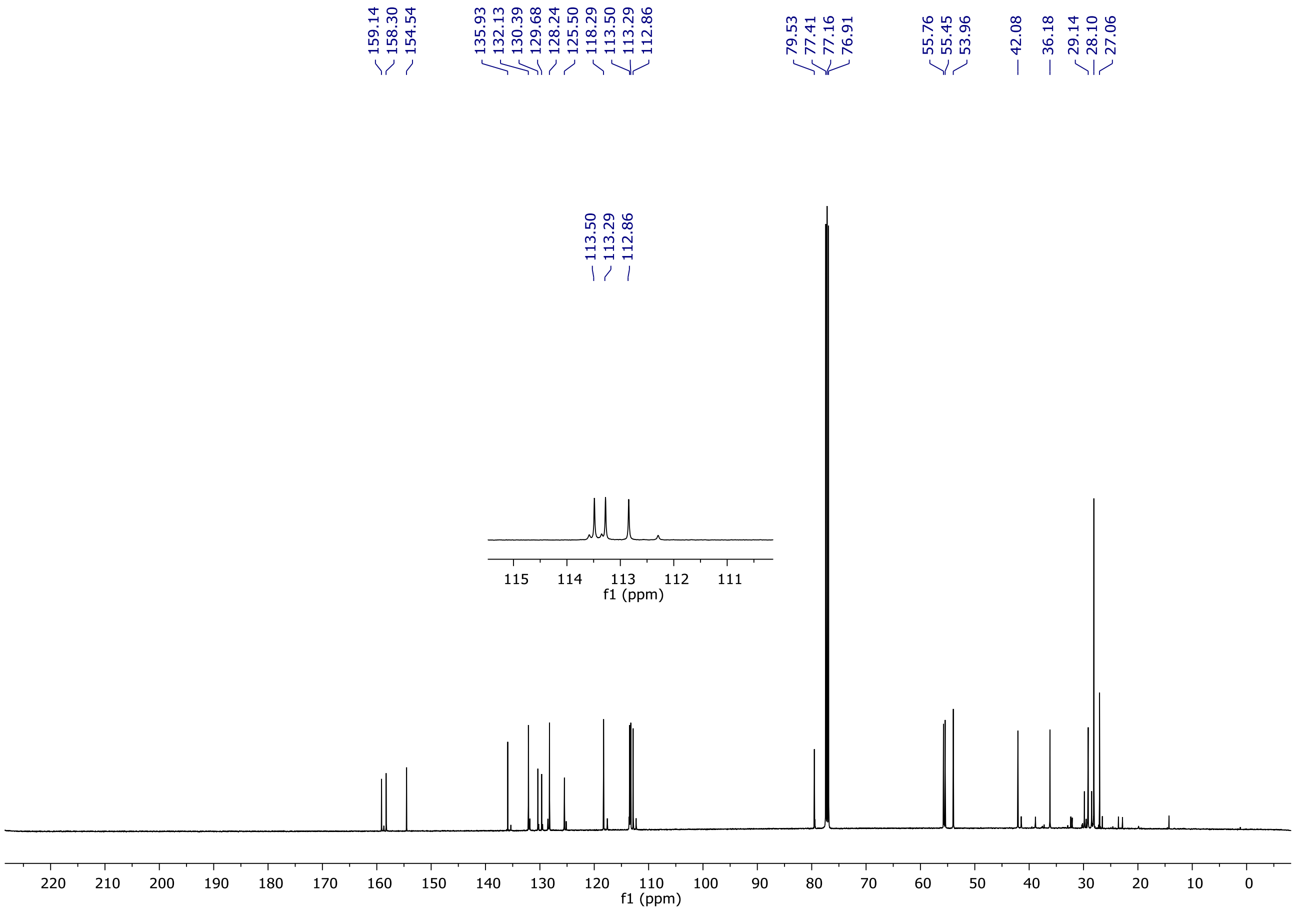
159.14
158.30
154.54
135.93
132.13
130.39
129.68
128.24
125.50
118.29
113.50
113.29
112.86
79.53
77.41
77.16
76.91
55.76
55.45
53.96
42.08
36.18
29.14
28.10
27.06
113.50
113.29
112.86
115 114 113 112 111
f1 (ppm)
220 210 200 190 180 170 160 150 140 130 120 110 100 90 80 70 60 50 40 30 20 10 0
f1 (ppm)

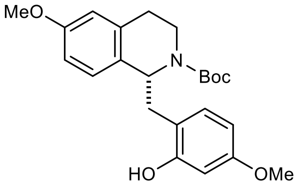
MeO
N
Boc
HO
OMe

19

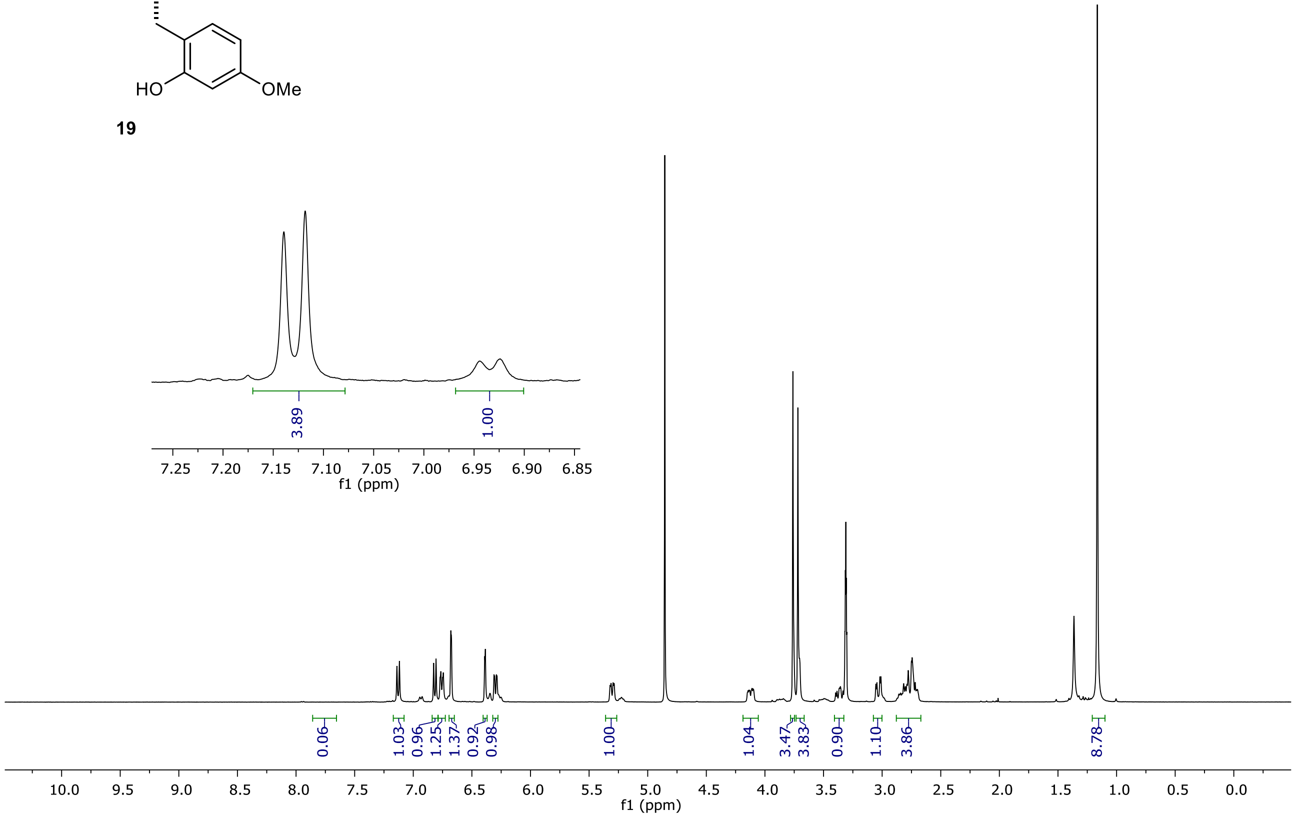
3.89
1.00
7.25 7.20 7.15 7.10 7.05 7.00 6.95 6.90 6.85
f1 (ppm)
0.06
1.03
0.96
1.25
1.37
0.92
0.98
1.00
1.04
3.47
3.83
0.90
1.10
3.86
8.78
10.0 9.5 9.0 8.5 8.0 7.5 7.0 6.5 6.0 5.5 5.0 4.5 4.0 3.5 3.0 2.5 2.0 1.5 1.0 0.5 0.0
f1 (ppm)

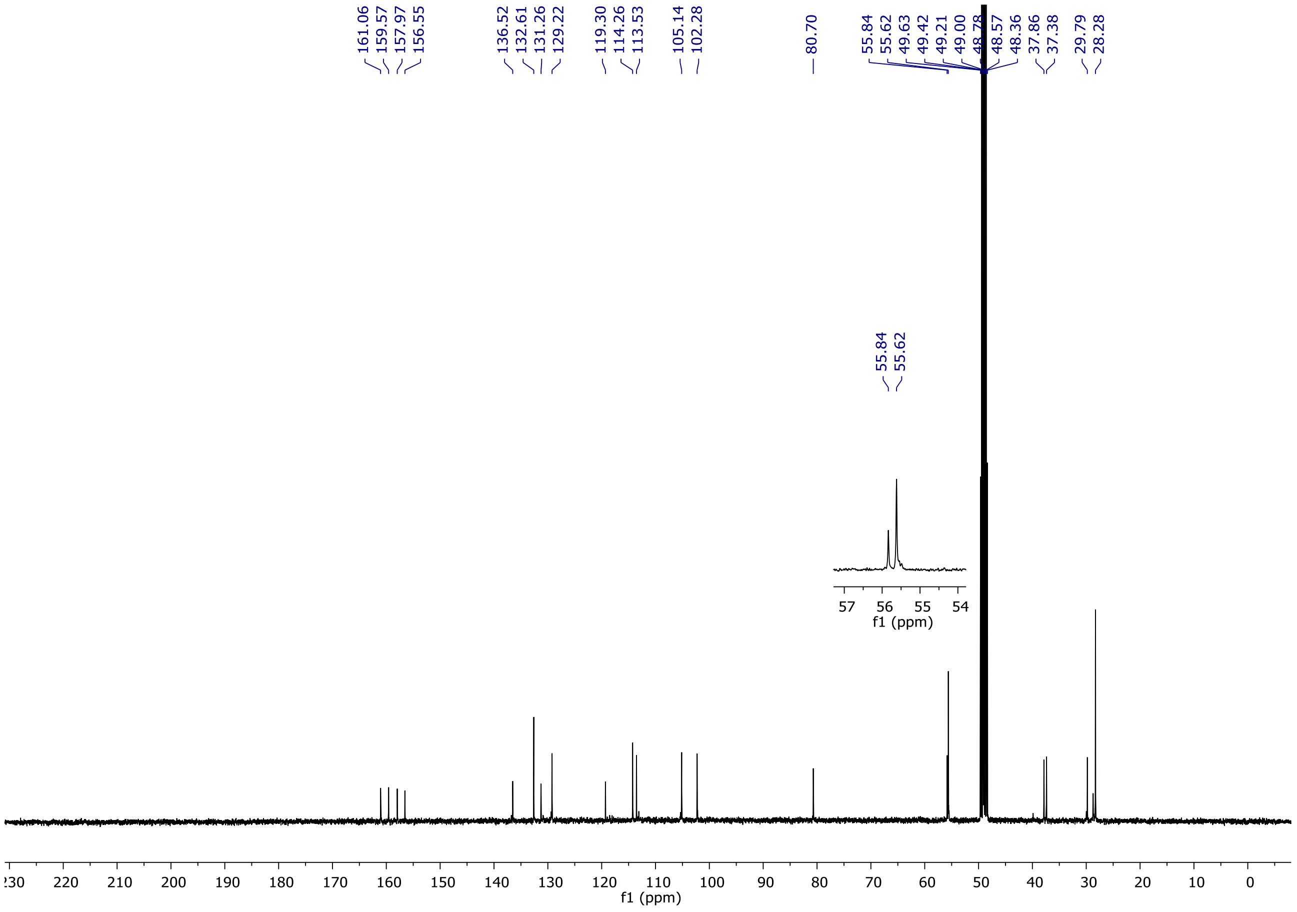
161.06
159.57
157.97
156.55
136.52
132.61
131.26
129.22
119.30
114.26
113.53
105.14
102.28
80.70
55.84
55.62
49.63
49.42
49.21
49.00
48.78
48.57
48.36
37.86
37.38
29.79
28.28
55.84
55.62
57 56 55 54
f1 (ppm)
230 220 210 200 190 180 170 160 150 140 130 120 110 100 90 80 70 60 50 40 30 20 10 0
f1 (ppm)

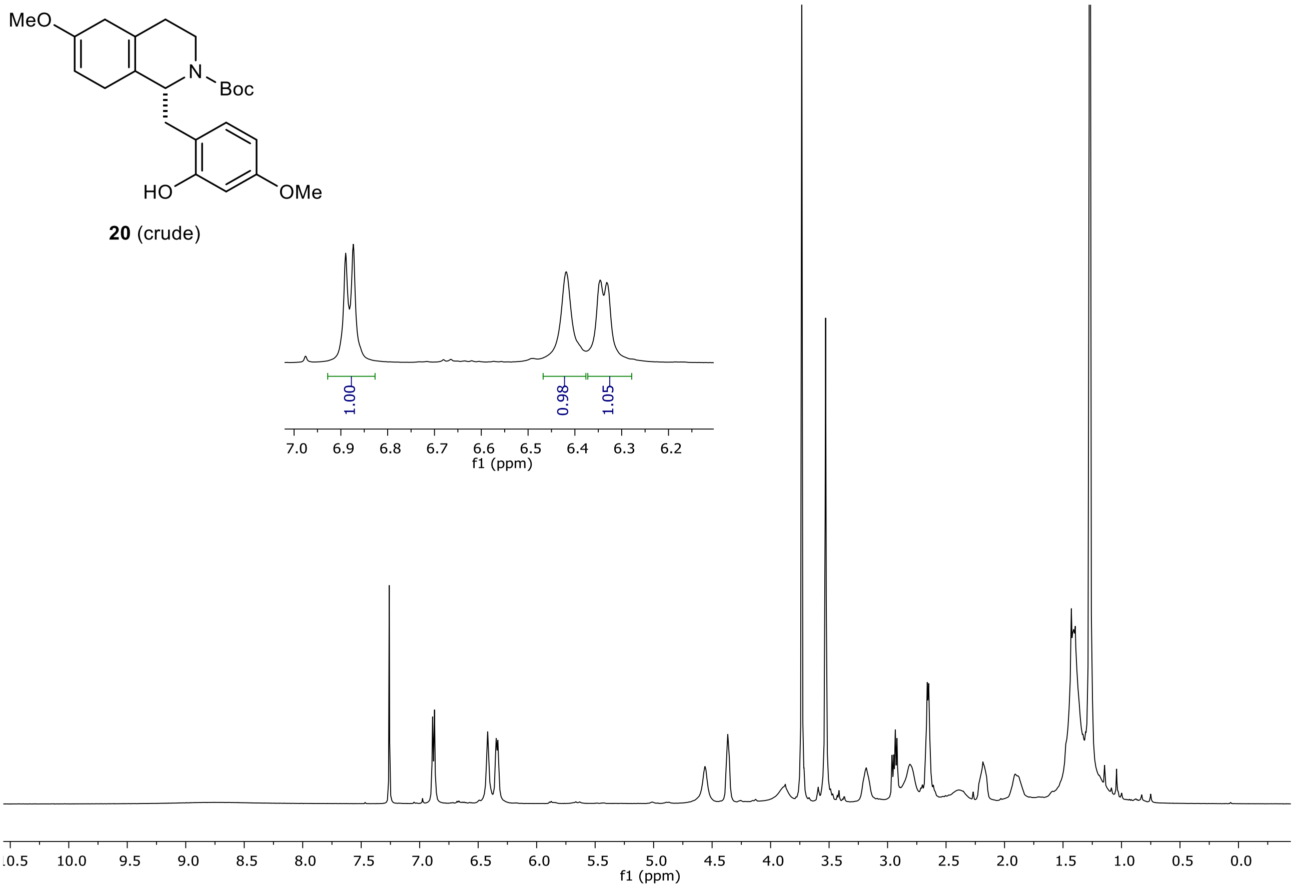

20 (crude)

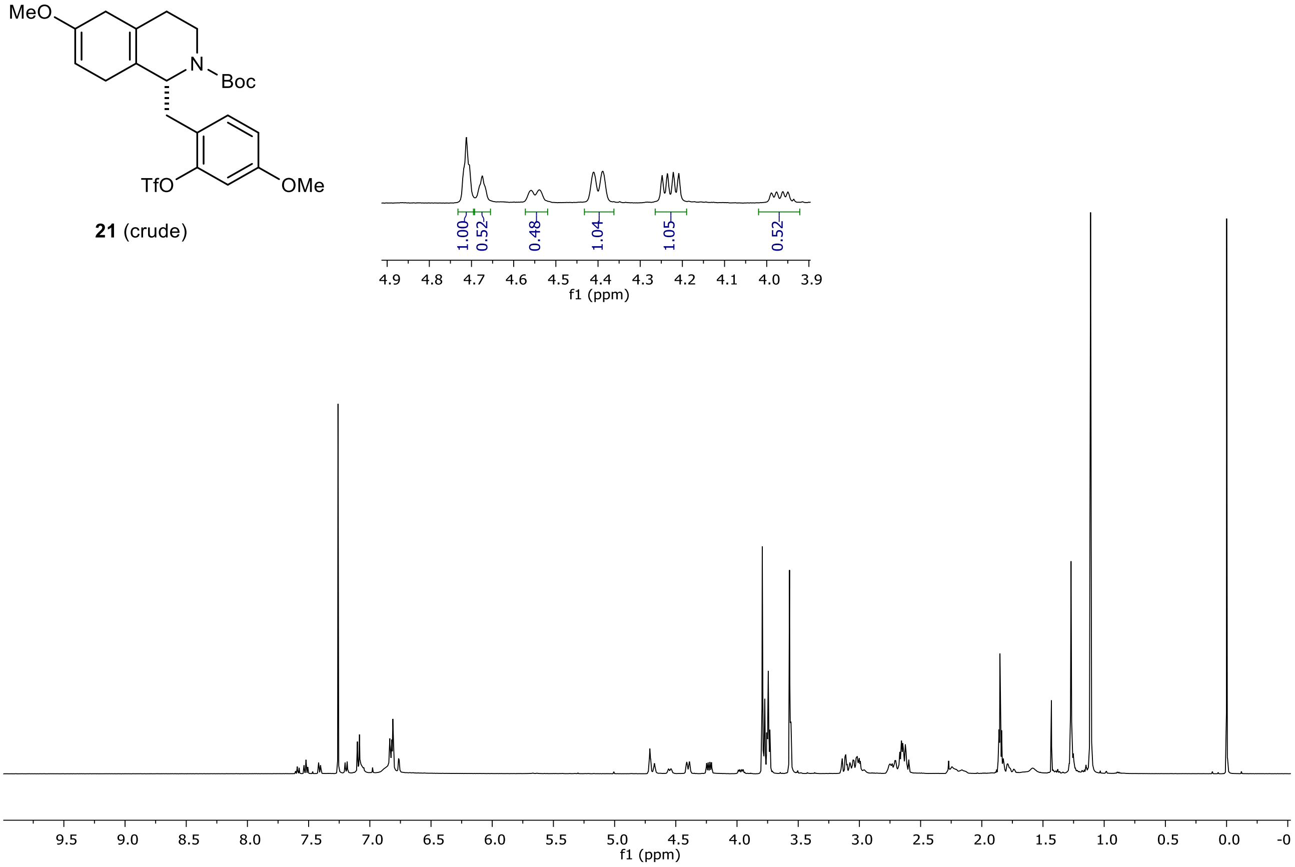

21 (crude)

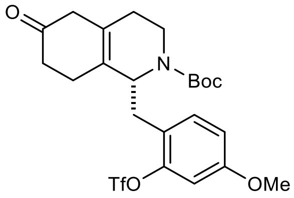

11 (crude)

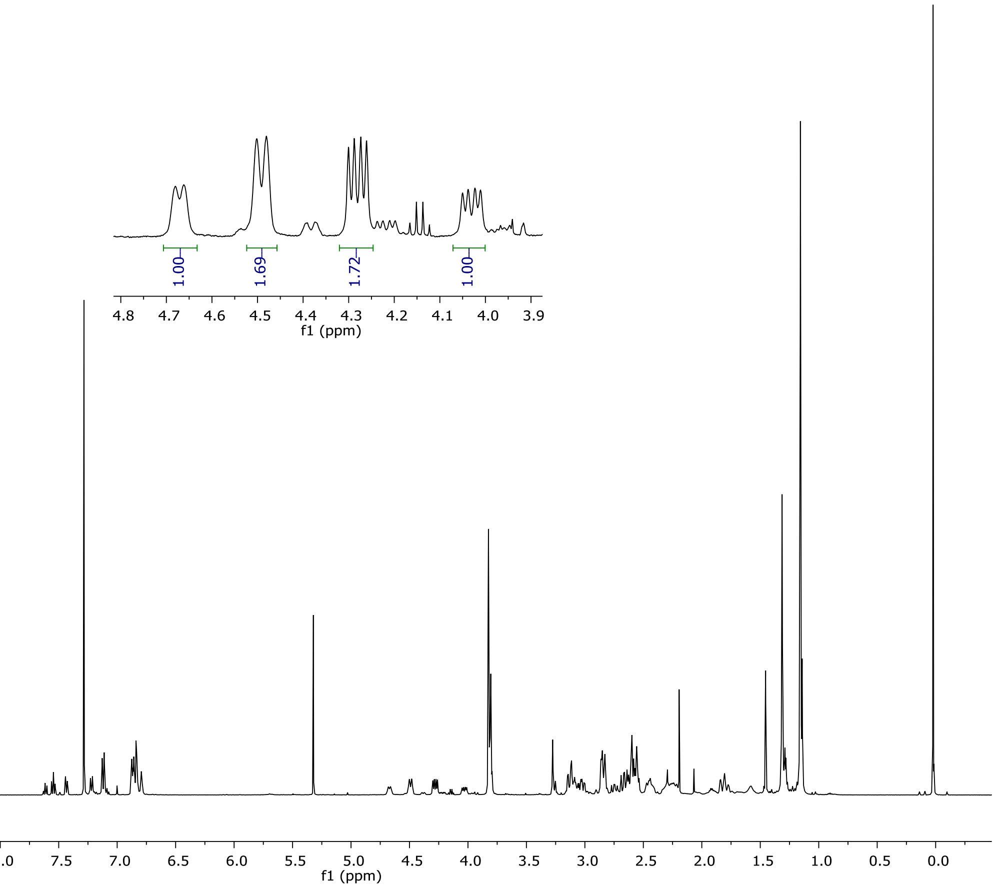
1.00
1.69
1.72
1.00
4.8 4.7 4.6 4.5 4.4 4.3 4.2 4.1 4.0 3.9
f1 (ppm)
0.5 10.0 9.5 9.0 8.5 8.0 7.5 7.0 6.5 6.0 5.5 5.0 4.5 4.0 3.5 3.0 2.5 2.0 1.5 1.0 0.5 0.0
f1 (ppm)

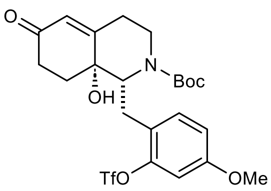
O
N
Boc
OH
TfO
OMe

22

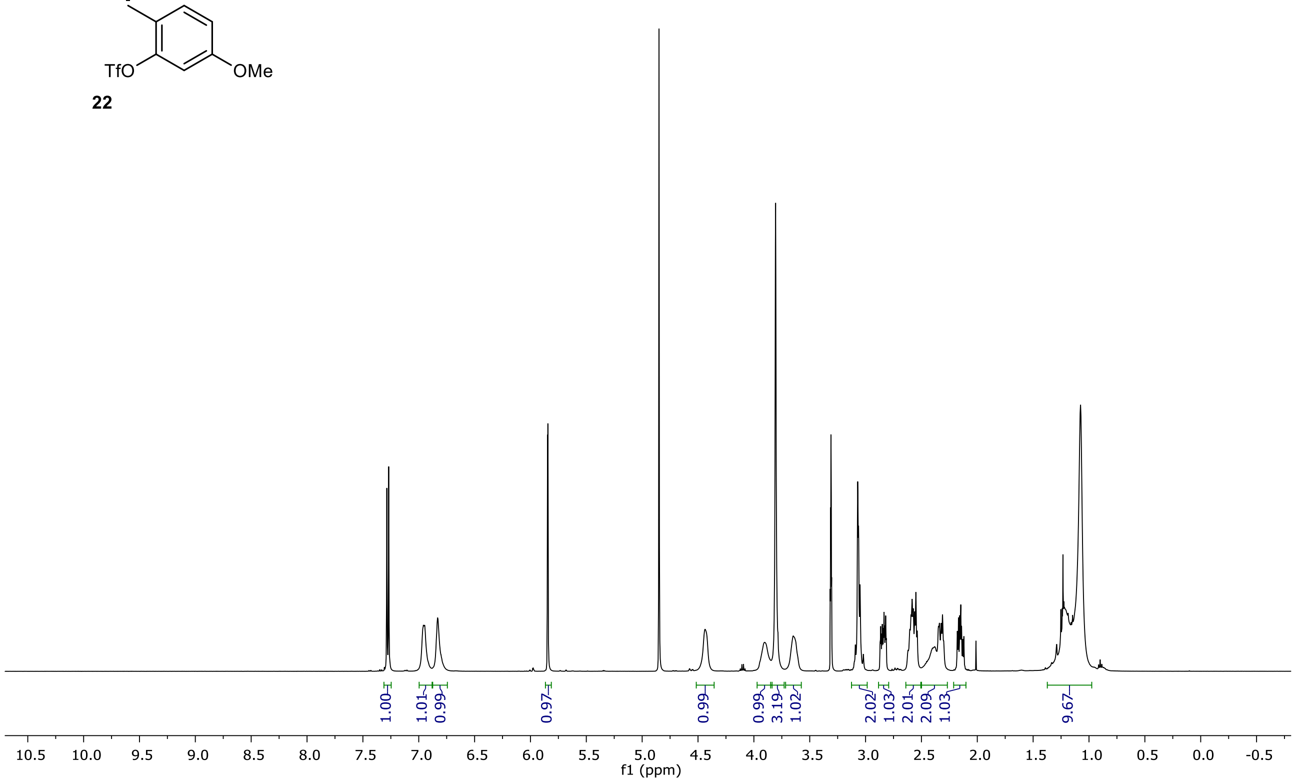
1.00
1.01
0.99
0.97
0.99
0.99
3.19
1.02
2.02
1.03
2.01
2.09
1.03
9.67
10.5 10.0 9.5 9.0 8.5 8.0 7.5 7.0 6.5 6.0 5.5 5.0 4.5 4.0 3.5 3.0 2.5 2.0 1.5 1.0 0.5 0.0 -0.5
f1 (ppm)

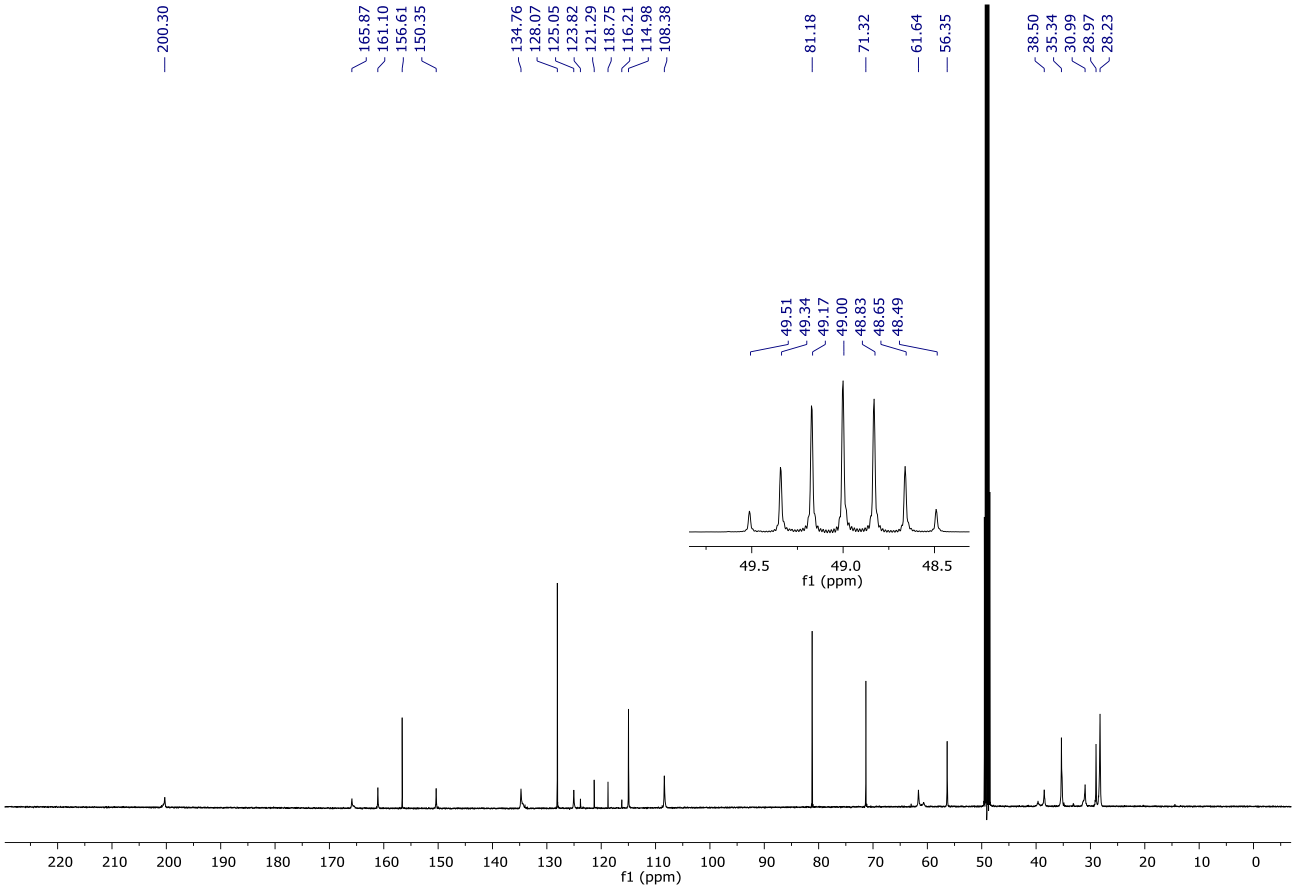
200.30
165.87
161.10
156.61
150.35
134.76
128.07
125.05
123.82
121.29
118.75
116.21
114.98
108.38
81.18
71.32
61.64
56.35
38.50
35.34
30.99
28.97
28.23
49.51
49.34
49.17
49.00
48.83
48.65
48.49
49.5 49.0 48.5
f1 (ppm)
220 210 200 190 180 170 160 150 140 130 120 110 100 90 80 70 60 50 40 30 20 10 0
f1 (ppm)

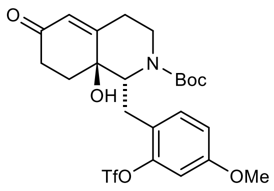
O
N
Boc
OH
TfO
OMe

12

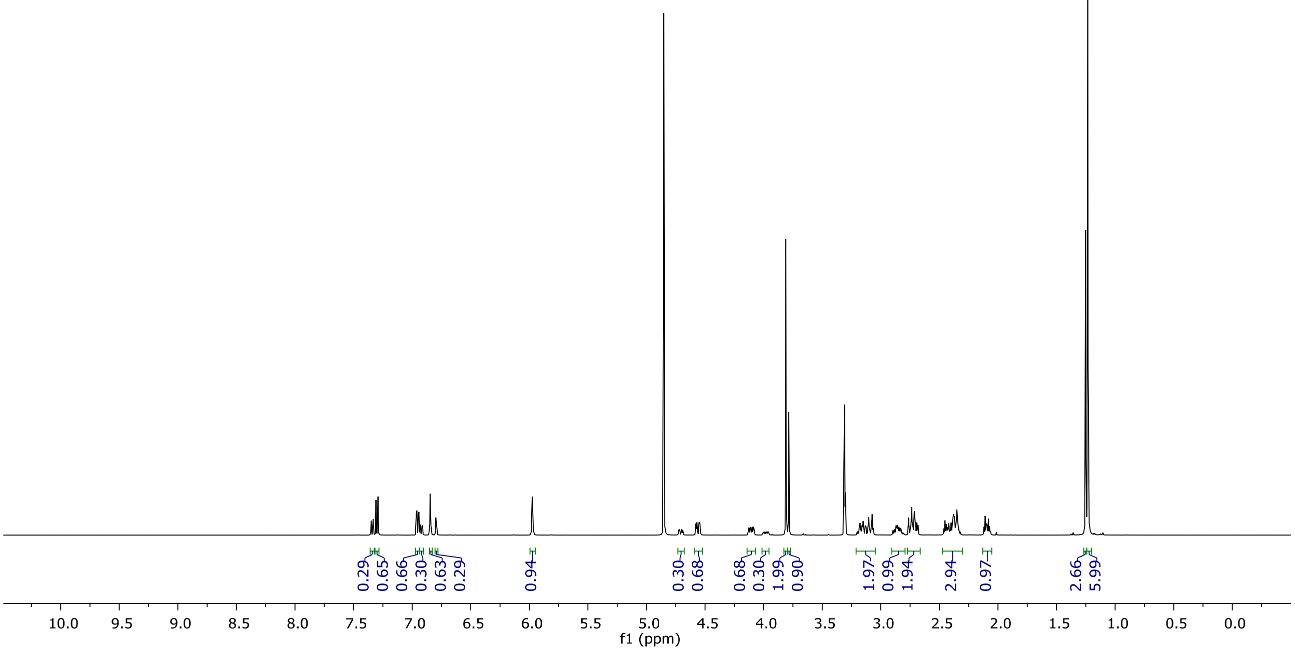
0.29
0.65
0.66
0.30
0.63
0.29
0.94
0.30
0.68
0.68
0.30
1.99
0.90
1.97
0.99
1.94
2.94
0.97
2.66
5.99
10.0 9.5 9.0 8.5 8.0 7.5 7.0 6.5 6.0 5.5 5.0 4.5 4.0 3.5 3.0 2.5 2.0 1.5 1.0 0.5 0.0
f1 (ppm)

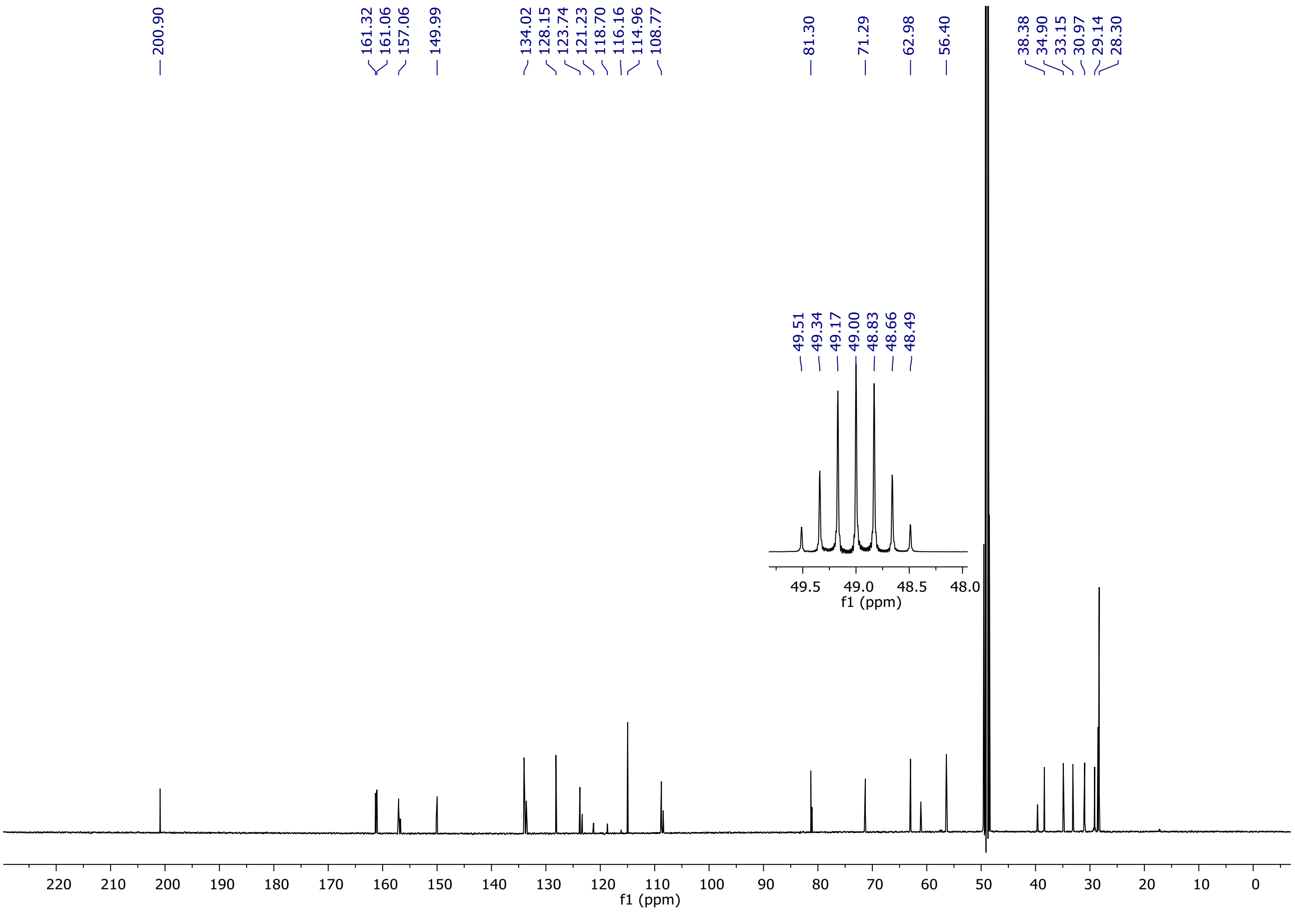
200.90
161.32
161.06
157.06
149.99
134.02
128.15
123.74
121.23
118.70
116.16
114.96
108.77
81.30
71.29
62.98
56.40
38.38
34.90
33.15
30.97
29.14
28.30
49.51
49.34
49.17
49.00
48.83
48.66
48.49
49.5 49.0 48.5 48.0
f1 (ppm)
220 210 200 190 180 170 160 150 140 130 120 110 100 90 80 70 60 50 40 30 20 10 0
f1 (ppm)

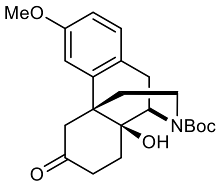
MeO
NBoc
OH
O

23

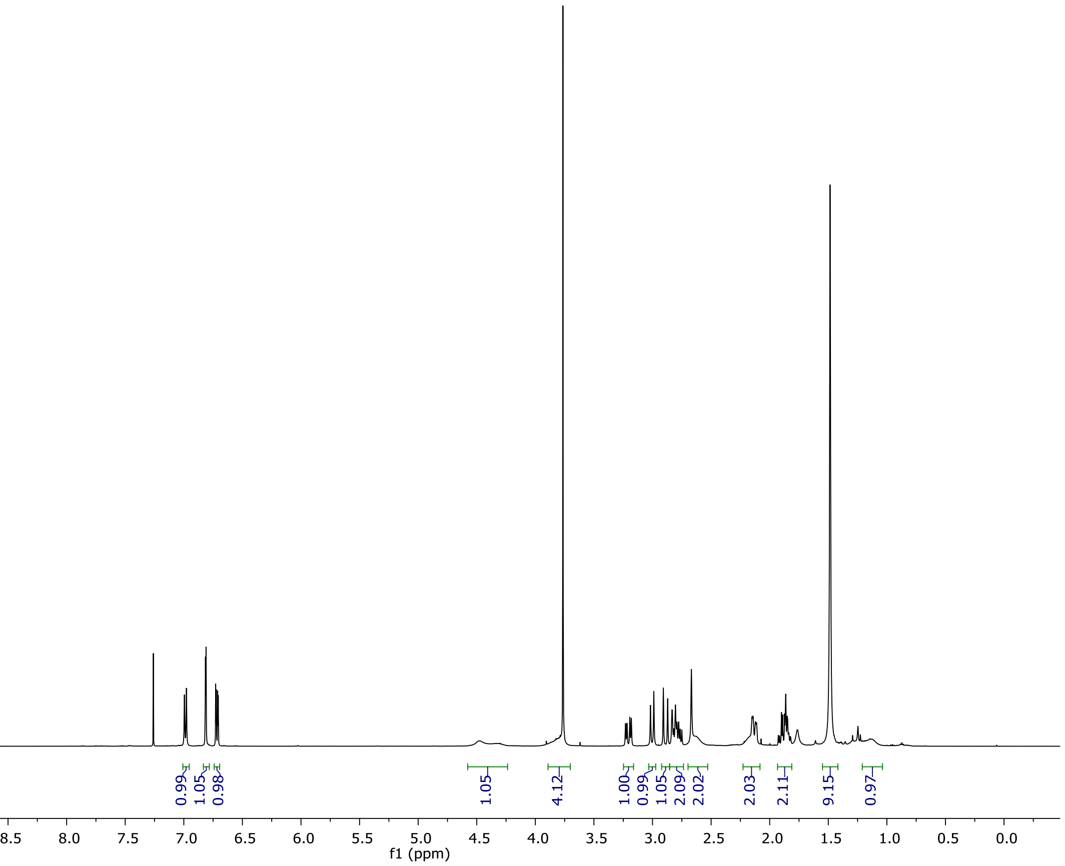
0.99
1.05
0.98
1.05
4.12
1.00
0.99
1.05
2.09
2.02
2.03
2.11
9.15
0.97
f1 (ppm)

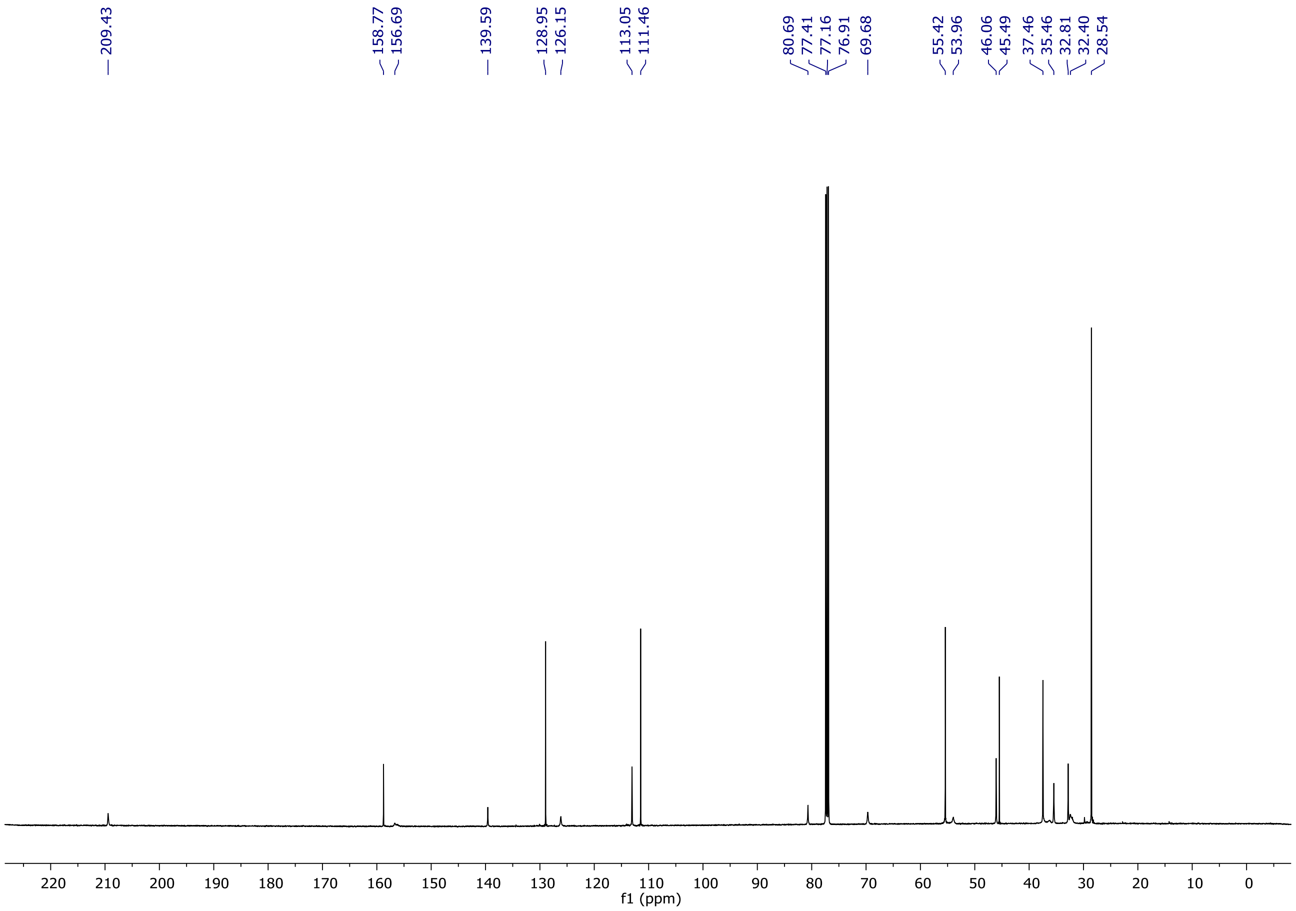
209.43
158.77
156.69
139.59
128.95
126.15
113.05
111.46
80.69
77.41
77.16
76.91
69.68
55.42
53.96
46.06
45.49
37.46
35.46
32.81
32.40
28.54
f1 (ppm)

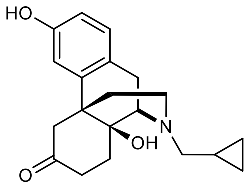
HO
N
OH
O

8

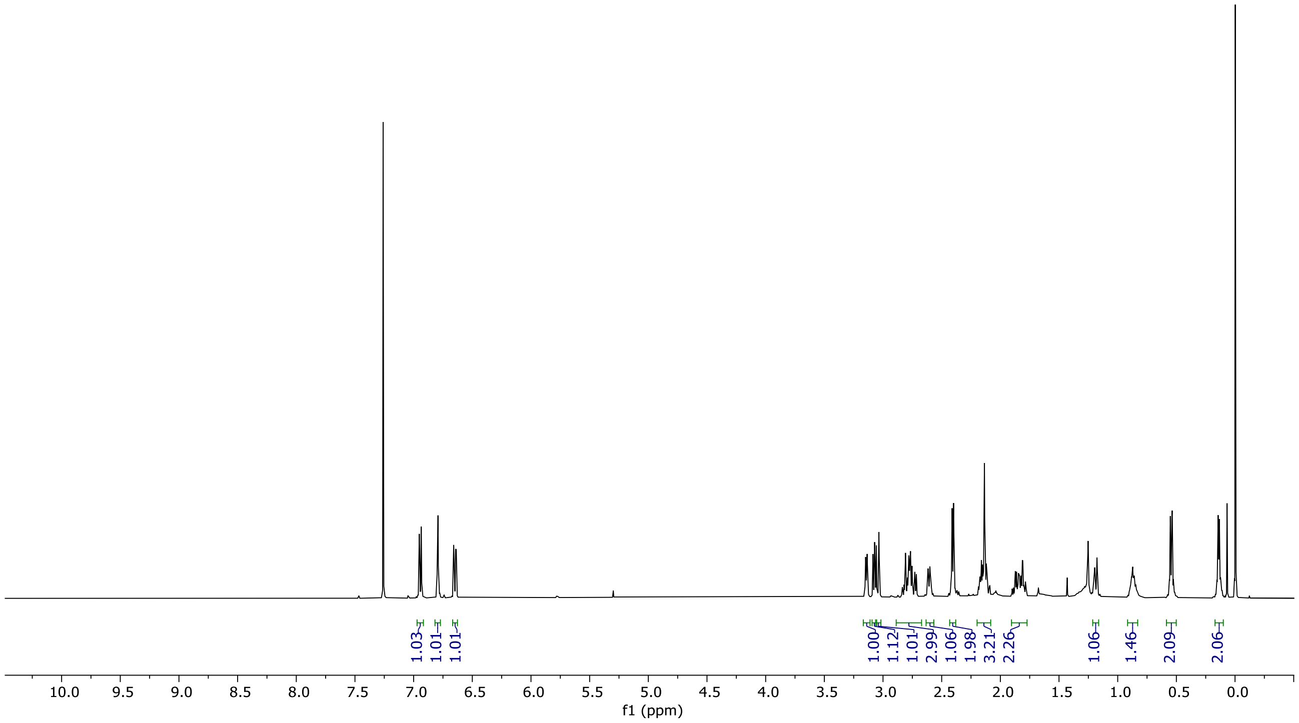
1.03
1.01
1.01
1.00
1.12
1.01
2.99
1.06
1.98
3.21
2.26
1.06
1.46
2.09
2.06
10.0 9.5 9.0 8.5 8.0 7.5 7.0 6.5 6.0 5.5 5.0 4.5 4.0 3.5 3.0 2.5 2.0 1.5 1.0 0.5 0.0
f1 (ppm)

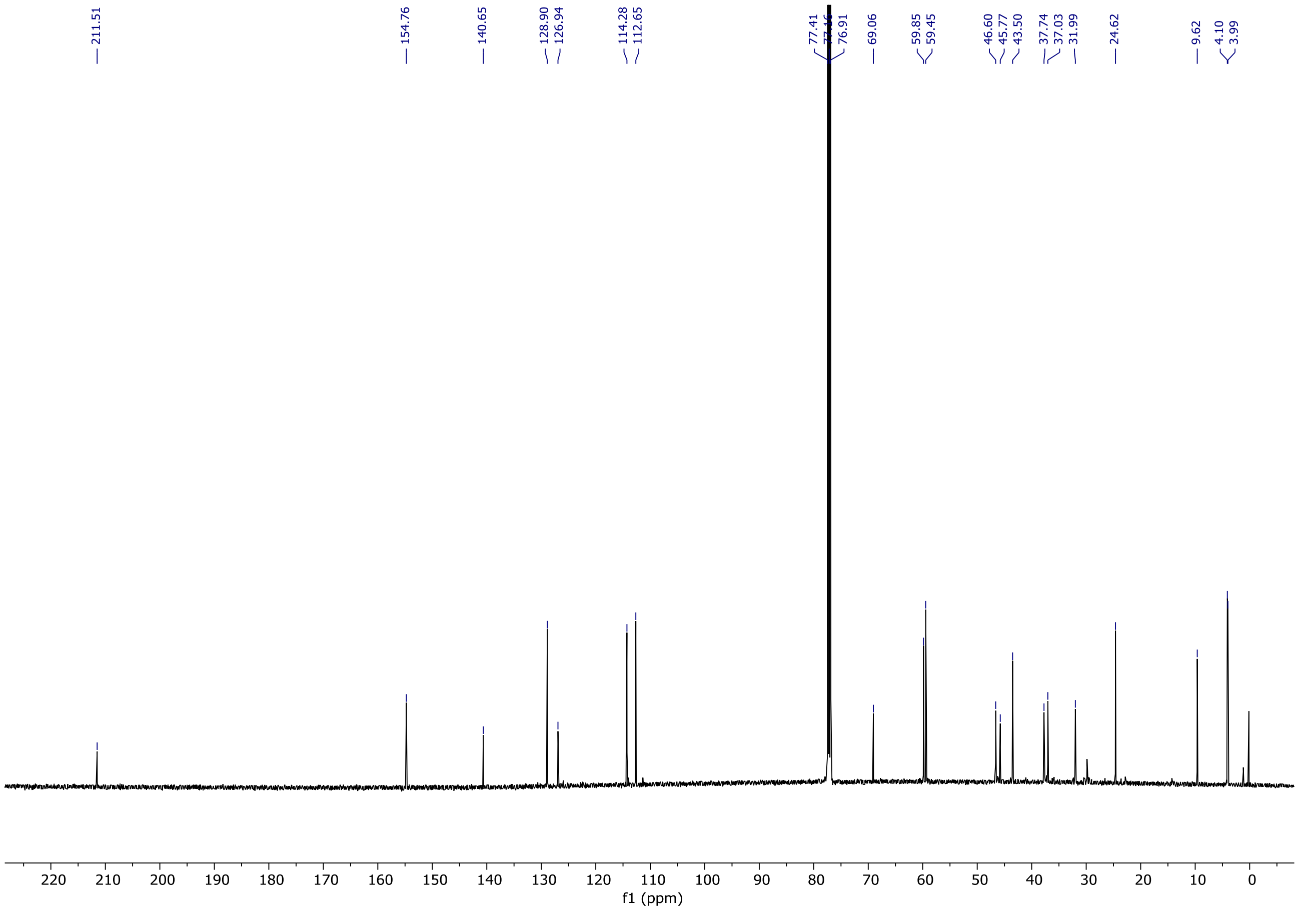
211.51
154.76
140.65
128.90
126.94
114.28
112.65
77.41
76.91
69.06
59.85
59.45
46.60
45.77
43.50
37.74
37.03
31.99
24.62
9.62
4.10
3.99
220 210 200 190 180 170 160 150 140 130 120 110 100 90 80 70 60 50 40 30 20 10 0
f1 (ppm)

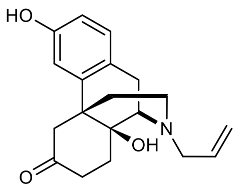

10

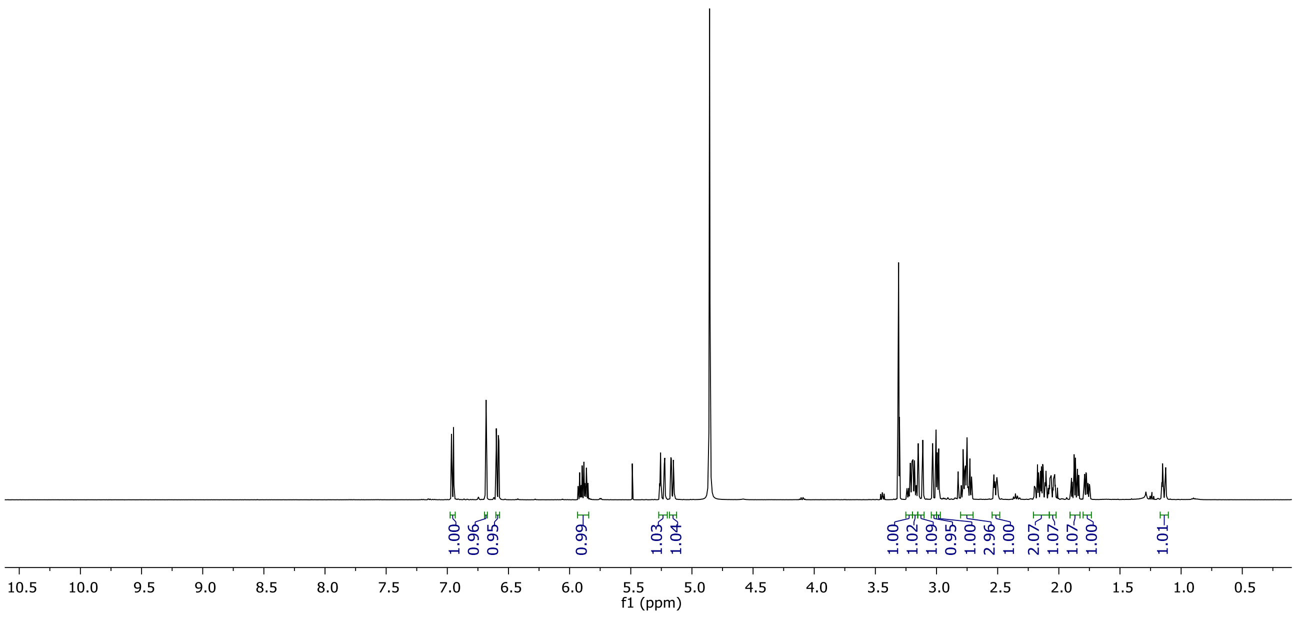
1.00
0.96
0.95
0.99
1.03
1.04
1.00
1.02
1.09
0.95
1.00
2.96
1.00
2.07
1.07
1.07
1.00
1.01
f1 (ppm)

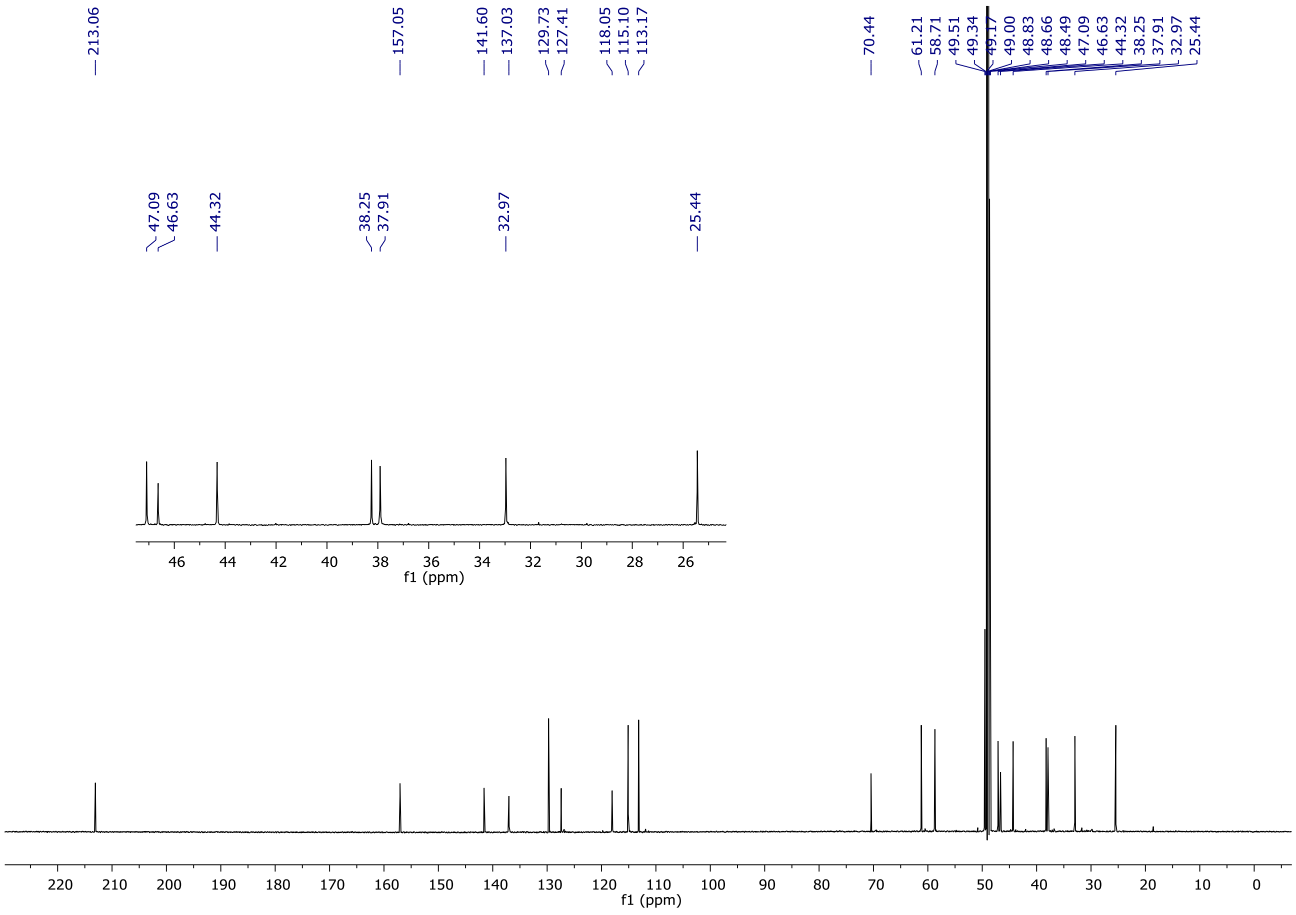
213.06
157.05
141.60
137.03
129.73
127.41
118.05
115.10
113.17
70.44
61.21
58.71
49.51
49.34
49.17
49.00
48.83
48.66
48.49
47.09
46.63
44.32
38.25
37.91
32.97
25.44
f1 (ppm)

6. HPLC Traces

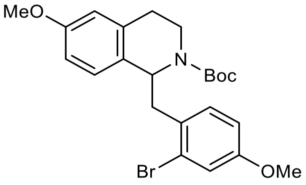

18 (racemic)

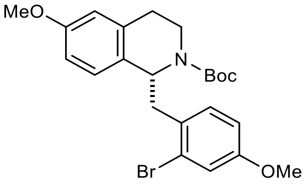

18 92% ee

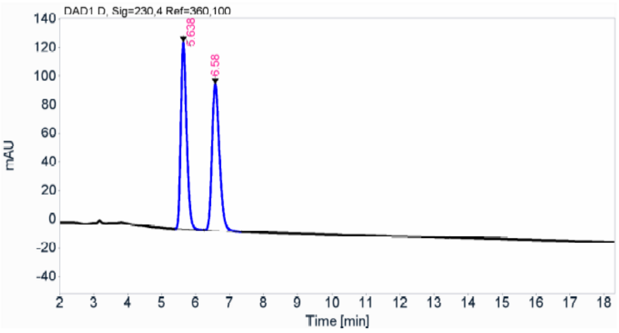

Signal: DAD1 D, Sig=230,4 Ref=360,100

RT [min]	Type	Width [min]	Area	Height	Area%
5.638	BB	0.1806	1552.396	130.6543	49.97
6.580	BB	0.2332	1554.201	102.3105	50.03

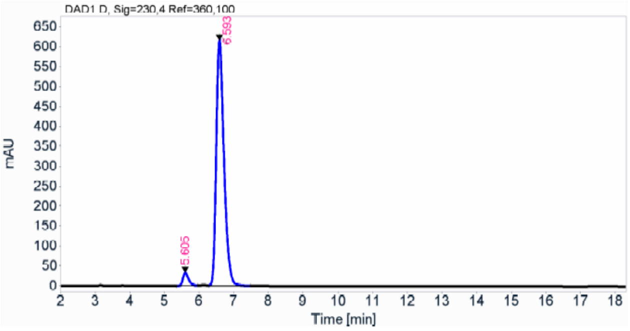

Signal: DAD1 D, Sig=230,4 Ref=360,100

RT [min]	Type	Width [min]	Area	Height	Area%
5.605	BB	0.1777	385.251	33.1099	3.89
6.593	VB	0.2361	9507.827	616.0742	96.11

7. References

1. J. Cosier, A. M. Glazer, *J. Appl. Crystallogr.* **1986**, *19*, 105-107.
2. Rigaku Oxford Diffraction, (2018), CrysAlisPro Software system, version 1.171.40.45a, Rigaku Corporation, Oxford, UK.
3. O. V. Dolomanov, L. J. Bourhis, R. J. Gildea, J. A. K. Howard, H. Puschmann, *J. Appl. Crystallogr.* **2009**, *42*, 339-341.
4. G. M. Sheldrick, *Acta Crystallogr. A* **2015**, *71*, 3-8.
5. G. M. Sheldrick, *Acta Crystallogr. C* **2015**, *71*, 3-8.
6. H. Mikula, D. Svatunek, D. Lumpi, F. Glöcklhofer, C. Hametner, J. Fröhlich, *Org. Process Res. Dev.* **2013**, *17*, 313-316.
7. B. R. Selfridge, X. Wang, Y. Zhang, H. Yin, P. M. Grace, L. R. Watkins, A. E. Jacobson, K. C. Rice, *J. Med. Chem.* **2015**, *58*, 5038-5052.

8 (a) T. H. El-Assaad, J. Zhu, A. Sebastian, D. V. McGrath, I. Neogi, K. N. Parida, *Org. Chem. Front.* **2022**, *9*, 5675-5725. (b) J. O. Edwards, R. H. Pater, R. Curclf, F. D. Furia, *Photochem. Photobiol.* **1979**, *30*, 63-70.

9. A. Bonifazi, F. O. Battiti, J. Sanchez, S. A. Zaidi, E. Bow, M. Makarova, J. Cao, A. B. Shaik, A. Sulima, K. C. Rice, V. Katritch, M. Canals, J. R. Lane, A. H. Newman, *J. Med. Chem.* **2021**, *64*, 7778-7808.
